# Should I Stay or Should I Go? Risk Perception and Use of Local Public Transport During the COVID-19 Pandemic

**DOI:** 10.3389/fpsyg.2022.926539

**Published:** 2022-07-07

**Authors:** Anna Helfers, Marissa Reiserer, Natalie Schneider, Mirjam Ebersbach, Carsten Sommer

**Affiliations:** ^1^Developmental Psychology, Institute of Psychology, Faculty of Human Sciences, University of Kassel, Kassel, Germany; ^2^Transportation Planning and Traffic Systems, Faculty of Civil and Environmental Engineering, University of Kassel, Kassel, Germany

**Keywords:** COVID-19, risk perception, public transport, mobility, pandemic resilience, protection motivation theory (PMT), climate change

## Abstract

In light of the climate crisis, the transport sector needs to be urgently transformed and the number of users of local public transport needs to be increased. However, the first year of the COVID-19 pandemic severely affected public transport with passenger numbers declining up to 80% in Germany. In addition to a general decrease in mobility during lockdowns, we can observe a shift in decision-making in regards to modes of transportation, with public transport losing out. We argue that this change in behavior can be explained by the fact that people tend to overestimate the risk of COVID-19 transmission in public transport. In order to understand risk perception in users and non-users of public transport during the pandemic, a representative survey (*N* = 918) in a German major city was conducted at the peak of the third wave of the pandemic in April 2021. We identified four main target groups of public transport use during the pandemic: Loyal users (*n* = 193), reducers (*n* = 175), pandemic-dropouts (*n* = 331) and non-users (*n* = 219). We found reducers (*r* = 0.12), pandemic-dropouts (*r* = 0.32) and non-users (*r* = 0.22) to perceive an increased perception of infection risk for public transport as compared loyal users. This increased risk perception was specific to public transport – it did not generalize to other day-to-day situations, such as going to the grocery store or visiting a hairdresser. This finding can be taken as an indication that risk perception for an infection plays a crucial role in stepping back from public transport use during the pandemic. In addition, however, there were other differences in terms of needs and concerns between the different target groups during the pandemic. Based on our findings, we discuss which tools and interventions might convince these different groups to hop-(back)-on public transport. Our study highlights how risk perception will play an important role in attracting new and former passengers and is the basis for the interventions and developments that will build a pandemic-resistant public transport in the future.

## Introduction

The German climate protection goals as named in the Federal Climate Change Act (Bundes-Klimaschutzgesetz, 2019; Hendzlik et al., 2021) require the German transport sector to decrease its greenhouse gas emissions by an equivalent of 85 million tonnes of CO_2_ by 2030 (Hendzlik et al., 2021). While the transport sector accounted for 20% of German greenhouse gas emissions in 2019, the envisioned target implies that the transport sector’s yearly emissions must be reduced by half within the next 8 years. This requires a fundamental transformation of the German traffic system. An expansion of local public transport (in the following: public transport) is one of the eight core-components of the German Federal Agencies’ action plan to realize this transformation (Hendzlik et al., 2021); Especially in urban areas where a great number of potential passengers could be transported on a relatively small amount of space, the advantages of local public transport could be played out against motorized individual mobility. In this way, public transport could play an important role in replacing cars. After all, the political aim prior to the pandemic was to double passenger numbers in public transport by 2030 (German Federal Government, 2018). The current coalition contract of the German Federal Government (2021) has an even more ambitious aim: to double the traffic performance by 2030, hence to double supply instead of doubling demand. This aim depends even more on an increase of passenger numbers.

However, since early 2020, the COVID-19 pandemic has been thwarting these ambitious plans: While public transport was considered critical infrastructure during the pandemic and was therefore provided on a constant level of around 80–100% (VDV, 2020), declines in its use by up to 80% (VDV, 2020) were observed, leaving buses and trains empty and abandoned. The resulting declines in revenues from ticket sales set public transport operators under financial pressure and consequently made governmental rescue packages necessary (Fedra, 2021; Verkehrsministerkonferenz, 2021). Today, after 2 years of the pandemic, passenger numbers in public transport have still not returned to 2019 levels (DLR Transport, 2022b). Public transport has lost about ten percent of their regular customers and a new normal – one with reduced public transport usage – seems to have established itself (DLR Transport, 2022b).

There are two main reasons for the word wide decline of public transport use during the pandemic. Firstly, a general reduction in the number and distance of trips among all modes of transportation (DLR Transport, 2022a,b); this general reduction can be considered a direct consequence of the lockdown (Askitas et al., 2021). Due to official regulations, people started working from home or were short time working, students did not go to school anymore, and social events of all sorts were canceled. People reported having generally fewer reasons to travel (DLR Transport, 2022b). In addition to this general decrease in mobility, however, a shift in the choice of transportation mode was observed, with public transport losing out (DLR Transport, 2022a). In the study of Finbom et al. (2020), participants from Germany reported a mode shift from public transport (bus, tram, and metro) to car, bicycling, and walking. In the study of Shibayama et al. (2021), a large shift from public transport to home office was noticed, but some participants also reported shifting to car and bicycling. In the April/May 2021 panel of DLR Transport, 53% of participants reported using public transport less or much less, whereas walking (+26%), bicycling (+18%), and car (+14%) usage had increased.

Protection motivation theory (Rogers, 1975; Floyd et al., 2000) suggests that risk perception might have played a role in these trends concerning the use of public transport: Since the beginning of the pandemic, public transport was stigmatized as an infectious space in public health communication campaigns (Tirachini and Cats, 2020). The perception of public transport as a high-risk environment might have triggered protection motivation in form of a general avoidance of traveling (Zheng et al., 2021) and a specific avoidance of public transport (e.g., Finbom et al., 2020; Shibayama et al., 2021; DLR Transport, 2022b). A positive relationship between risk perception and protective behavior is already known from other pandemics and infectious disease outbreaks (for a review, see Bish and Michie, 2010). Regarding the COVID-19 pandemic, empirical evidence suggests that risk perception determines hygiene and social distancing behavior (Majid et al., 2020; Wise et al., 2020). Indeed, when asked about their COVID-19 related risk perceptions, people reported feeling much more inconvenient in public transport since the beginning of the pandemic, but not when bicycling and walking (DLR Transport, 2022a,b). While the car was perceived as a safe space, metro, tram, train and bus were perceived as high-risk environments (Finbom et al., 2020). In the survey of DLR Transport (2022b), former public transport users spoke of the hygiene in the vehicles and the inability to keep their distance from other passengers as the two main reasons for deciding against using buses and trams within the pandemic. Shibayama et al. (2021) found that 72% of those who shifted from public transport to other travel modes reported to do so because of an increased risk perception in public transport. A more differentiated look on users and non-users of public transport during the pandemic revealed an interesting pattern. Whereas ongoing public transport users perceived themselves at greater susceptibility of infections than non-users in the study of Costa (2020); Finbom et al. (2020) report a reverse relationship. In their study, those who avoided using public transport during the COVID-19 pandemic reported to perceive an increased infection risk during public transport use as compared to other day-to-day situations (e.g., going to a warehouse) and as compared to the ones who continued using public transport. These contradictory findings might in part be explained by the fact, that – of course – not all individuals have the opportunity, in terms of means and finances, to perform protective behaviors in response to risk perception, such as avoiding public transport (d’Arbois de Jubainville and Vanier, 2017). Finbom et al. (2020) showed that education level and the working situation, as well as income, affected transport mode choice during the pandemic. People with lower income reported lower access to alternatives to public transport. In the same vein, in the study of Shibayama et al. (2021) those who continued to use public transport reported to do so, because they had no other opportunity.

This increased risk perception for public transport stands in contrast to considerations (Sommer et al., 2021), that the objective infection risk is rather low in public transport: Two thirds of trips in public transport have a duration of less than 15 min, making contact to others very brief (Sommer et al., 2021). Efficient fresh air ventilation systems (Greenhalgh et al., 2022) and mask wearing protects very well against infections (Chu et al., 2020; Ueki et al., 2020; Bagheri et al., 2021) and has become the new norm in public transport. Moreover, initial empirical findings have also reported no increased risk of infection for users of public transport (Charite Research Organisation [CRO], 2021; Galmiche et al., 2021).

In our study, our goal was to investigate the impact of COVID-19 risk perception on public transport use in a representative sample of a German major city. As reported, there is already a lot of research regarding COVID-19 and public transport (e.g., Finbom et al., 2020; Shibayama et al., 2021; DLR Transport, 2022a,b). However, most of the existing evidence on risk perception stems from samples recruited *via* snowball sampling (Shibayama et al., 2021), involving open online questionnaires, in which self-selection processes could have distorted the results, or the surveys included (former) public transport subscribers only (e.g., Finbom et al., 2020; Charite Research Organisation [CRO], 2021). We aimed to extend these findings by focusing on a representative sample for a German major city, allowing us to draw general conclusions concerning risk perception and its effects on public transport use in urban areas and recommend strategies for bringing people back to public transport after the pandemic. First, we quantified changes in mobility patterns in the representative sample taken during the pandemic. We assessed the frequencies of transport mode usage and mode shifts during the pandemic to determine how many people continued using public transport during the pandemic, how many reduced or dropped out of public transport during the pandemic, and how many were general non-users. We next wanted to understand to what extent public transport fulfilled different needs and concerns of mobility during the pandemic. Therefore, we evaluated infection risk perceptions for public transport, but also determined which criteria were generally perceived as relevant for the choice of transport mode during the pandemic, to what extent they were fulfilled in public transport and which measures meant to prevent COVID-19 transmission in public transport were perceived as important. To understand to what extent these needs and concerns determined transport mode choice during the pandemic, we looked at differences between the four target groups regarding these needs and concerns. Thereby, we aimed to identify leverage points to bring people back to public transport after the pandemic.

## Materials and Methods

### Data Collection

An online questionnaire implemented with Lime Survey (Lime Survey Community Edition, version 3.27.19) was applied. The questionnaire was provided in German, English, Turkish, and Russian. For people who had difficulties with this online format the option of telephone participation was offered.

### Participants

A representative sample of 3000 people aged between 14 and 85 years was drawn from all residents of a German major city. The drawing was conducted by the city’s residents’ registration office. All selected individuals were invited to participate *via* an individualized letter sent to their private address, containing a one-euro piece as incentive for participation. The survey was conducted during the second lockdown in Germany (Kodzo and Imöhl, 2022), between April 21st 2021 and May 12th 2021. During this period, the local 7-day COVID-19 incidence ranged between 152 and 234 per 100,000 residents and the vaccination rate was still low, increasing from 21 to 36% during the survey period (Statista, 2022). At the end of the first week of the survey, the previously discussed and announced measure of mandatory wearing of an FFP2 mask in public transport was implemented by law (Federal Ministry of Health, 2021). During the survey period, the online questionnaire was accessed 1065 times. After excluding inadmissible values (participation code not assignable: *n* = 9; did not finish the survey or skipped more than 15% of questions: *n* = 130, no information in public transport mode change *n* = 8) the final data set consisted of *N* = 918 participants. This corresponds to a response rate of 31%. Participants (*n_female_* = 465; *n_male_* = 453; *n*_diverse_ = 0) were aged between 14 and 86 years (*M* = 47.64, *SD* = 18.57). A detailed overview over socio-demographics can be found in Table 1.

**TABLE 1:** Sociodemographic characteristics of participants.

			Target Groups							
	Total *N* = 918		Loyal *n* = 193		Reducers *n* = 175		Dropouts *n* = 331		Non-users *n* = 219	
	*n*	%	*n*	%	*n*	%	*n*	%	*n*	%
**Gender**										
Female	465	51	96	50	98	56	170	51	101	46
Divers	0	0	0	0	0	0	0	0	0	0
Male	453	49	97	50	77	44	161	49	118	54
Occupation*										
Employed	495	54	80	41_D_	84	48_D_	200	60_L,R_	131	60
Not employed	38	4	4	2	8	5	13	4	13	6
Retired	210	23	50	26	37	21	70	21	53	24
Student	142	15	55	39_D_	38	27_D_	34	24_L, R_	15	11
Care work	23	3	3	2	7	4	11	3	2	1
**Language**										
German	913	99	192	99	175	100	329	99	217	99
English	1	0	0	0	0	0	0	0	1	0
Russian	4	0	1	1	0	0	2	1	1	0
**Financial situation**										
Very stable	384	42	73	38	63	36	148	45	100	46
Stable	315	34	68	35	69	39	114	34	64	29
Rather stable	152	17	37	19	29	17	51	15	35	16
Rather precarious	43	5	7	4	13	7	14	4	9	4
Precarious	8	1	4	2	1	1	1	0	2	1
**Vaccinated against COVID-19**										
Yes	247	27	53	27	40	23	86	26	68	31
No	622	68	131	68	129	74	226	68	136	62
No response	49	5	7	4	4	2	14	4	11	5
**Risk Group**										
Yes	319	35	70	36	59	34	114	34	76	35
No	424	46	90	47	88	50	142	43	104	47
No response	175	19	31	16	26	15	73	22	37	17
**Already infected with COVID-19**										
Yes	35	4	11	6	7	4	10	3	7	3
No	844	92	176	91	163	93	306	92	199	91
No response	39	4	3	2	3	2	11	3	10	5
Dependance on PT*										
Reliant on PT	134	15	83	43_R, D, N_	44	25_L, D, N_	6	2_L, R_	1	0_L, R_
Alternatives but PT preference	140	15	69	36_R,D, N_	40	23_L, D, N_	22	7_L, R_	9	4_L, R_
Alternatives and preferred	408	44	27	14_R, D, N_	69	39_L, D, N_	207	63_L, R_	105	48_L, R_
PT not accessible	231	25	14	7_R, D, N_	21	12_L, D, N_	95	29_L, R_	101	46_L, R_
**Ticket**										
Single or short trip ticket	86	9	36	19	50	29	NA	NA	NA	NA
Group ticket	32	3	19	10	13	7	NA	NA	NA	NA
Weekly/monthly pass, no subsc.	19	2	11	6	8	5	NA	NA	NA	NA
Annual/monthly pass. with subscr.	33	4	18	9	15	9	NA	NA	NA	NA
Semester/School/Seniors Ticket	110	12	59	31	51	29	NA	NA	NA	NA
Job ticket, BC100, handicapped ID	72	8	42	22	29	17	NA	NA	NA	NA
Never use public transport	1	0	1	1	0	0	NA	NA	NA	NA
Other	36	4	7	4	9	5	NA	NA	NA	NA

PT, public transport. subsc., subcription. *p < 0.006 (Bonferroni-corrected for the nine omnibus tests) in the omnibus Chi-squared test. Pairwise group comparisons (Bonferroni-corrected p_crit_ = 0.008) are based on pairwise two-sided Pearson’s Chi-squared test with Yates’ continuity correction. For each significant difference between the target group in the column header and the other target group(s), the letter of the significantly different group (L, loyal users; R, reducers; D, dropouts; N, non-users) appears as subscript.

### Measurements

The questionnaire was structured in three parts. In the first part, participants were asked about their current mobility patterns and reported which factors generally influence their mode choice of transport. In the second part, participants evaluated several occupation scenarios of vehicles. This part of the questionnaire will be analyzed in a separate paper and is therefore not further considered here. COVID-19 related questions on perception of infection risk and the evaluation of the different measures were addressed in the third part. The questionnaire ended with a section on socio-demographic data. On average, the participants needed 25 min to complete all questions.

#### Frequency and Modes of Transportation Used During the Pandemic

The frequency of using different means of transportation during the pandemic was evaluated on an item that was taken from Nobis and Kuhnimhof (2018): “When thinking about the last month, how often did you use the following means of transportation?”. Participants reported the frequency of having used the transportation modes walking, bicycling, pedelec, bus, tram, train, car – driving, car – passenger, motor bike on an ordinal scale with the response options “I do not have access to this mode,” “seldom or never,” “1 to 3 days per month,” “1 to 3 days per week,” and “(almost) daily.”

#### Transport Mode Shift During the Pandemic

Mode shift during the pandemic was assessed with the item “Did the COVID-19 pandemic influence your transport mode choice? Due to the COVID-19 pandemic I use…”. Participants evaluated their change in using the transport modes walking, bicycling, pedelec, bus and tram, car – driving, car – passenger, motor bike, from before the pandemic to during the pandemic on an ordinal scale with the response options “much decreased,” “decreased,” “no change,” “increased,” and “much increased.”

#### Infection Risk Perception

Participants were asked to evaluate their perceived infection risk for ten different day-to-day situations. In addition to public transport (using bus and tram), the day-to-day situations going to the grocery store, going to work, going to school, meeting friends at home, going out for a walk, going to the hairdresser, and taking a cab were selected. Participants were asked to evaluate the perceived infection risk of these specific situations on a five-point Likert-scale ranging from 1 (very low) to 5 (very high).

#### Criteria of Transport Mode Selection

To understand participants’ general criteria when it comes to transport mode selection, we applied the multiple-selection item “We would like to know the reasons for which you choose of the above-mentioned means of transport: Which factors are important to you when choosing your means of transport?” The following response options were presented in a randomized order: accessibility, comfort, costs, ease of planning, ease of use, flexibility, fun, privacy, protection against accidents, protection against infections, protection against harassment, discrimination and violence, reachability, reliability, sustainability, travel duration, weather, other.

#### Satisfaction With These Criteria in Public Transport

In a next step, participants were asked to evaluate to what extent these criteria were fulfilled in public transport. Therefore, the fulfilment of each of the criteria named above was evaluated on a five-point Likert-scale ranging from 1 (very bad) to 5 (very well) fulfilled in public transport.

#### Evaluation of Measures Taken in Public Transport to Decrease the Infection Risk

Finally, we presented 16 measures taken to reduce the objective infection risk in public transport during the pandemic. The measures were the result of our systematic research on measures in public transport that had already been implemented or were discussed up to the time of the survey. They comprised policy measures within the vehicle, such as mandatory mask wearing, and entrance only with a negative test, digital measures such as an advance occupation information tool and contactless ticket purchase, measures to maintain distance within the vehicle, such as blocked seats and protective screens, but also service and infrastructural measures such as providing disinfectants and masks. We asked: “Which of these measures would have to be implemented in order to enable you to use public transport under pandemic conditions with a good and safe feeling?” Each of the presented measures was evaluated by the participants on a five-point Likert-scale ranging from 1 (very unimportant) to 5 (very important).

#### Socio-Demographic Data

Participant’s gender, age, their financial and occupational status were assessed as demographic data. These questions were based on the questionnaire from Finbom et al. (2020). We furthermore assessed if participants or their household members belonged to a risk group, asked about their current vaccination status and whether they had already been infected with COVID-19. We also evaluated to what extent participants could choose between different modes of transportation and which ticket they usually selected.

#### Data Elaboration

**FIGURE 1:**
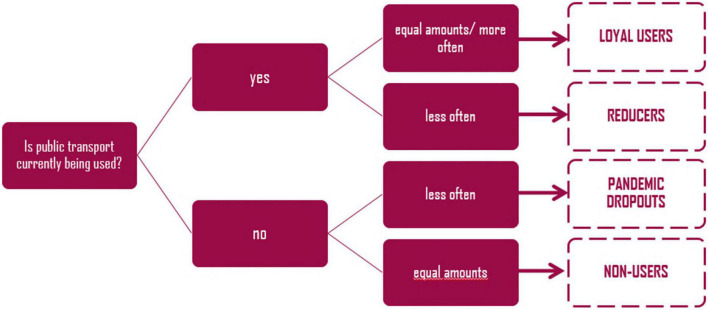
Target groups of public transport use during the pandemic.

Data sets including non-assignable code (*n* = 9), more than 15% of skipped questions (*n* = 130), or no information concerning the variable transport mode use or transport mode shift (*n* = 8) were coded invalid data and excluded from the dataset, resulting in a final sample of *N* = 918. Based on the two items for transport mode use during the pandemic and transport mode shift from before the pandemic, we computed the variable target group. In a first step, participants who reported currently using public transport (i.e., bus or tram) “(almost) daily,” “1 to 3 days per week,” or “1 to 3 days per month,” were coded as “ongoing public transport users.” Participants who reported using public transport “seldom or never,” and those having “no access” to this mode were coded as current “non-users.” In a subsequent step, those “ongoing public transport users” who reported having “strongly increased,” or “increased” the use of bus and tram or reported “no change,” were coded as “loyal users” (*n* = 193). Ongoing public transport users who reported having “decreased,” or “strongly decreased” the use of bus and trams during the pandemic were coded as “reducers” (*n* = 175). In turn, current “non-users” who reported having “decreased,” or “strongly decreased” the use of bus and trams during the pandemic were coded as “pandemic “dropouts” (*n* = 331), and those who reported “no change” were coded as “non-users” (*n* = 219) (see also Figure 1).

#### Data Diagnostics

In the case of missing values, the list-wise case exclusion was applied. Therefore, only cases were considered in which all variables involved had valid expressions. Therefore, the number of cases evaluated varies depending on the variable.

#### Statistical Procedure

Statistical analyses were performed with R Studio (Version 1.4.1717). When more than two groups were compared, the significance level (*p_crit_* = 0.05) was adjusted using Bonferroni correction.

## Results

### Transport Mode Use Before and During the Pandemic and Target Groups

**FIGURE 2:**
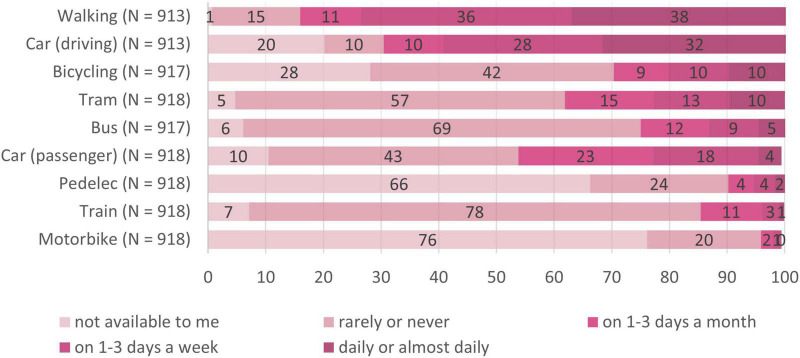
Means of transportation used in the last month. *N* = 918; numbers on the bars represent percentages.

Frequency and mode of transportation used during the pandemic are illustrated in Figure 2. Looking at public transport, 38% of the participants reported having used the tram and 25% the bus at least 1 to 3 days per month within the last month. Only 10% used the tram and 6% used the bus daily.

**FIGURE 3:**
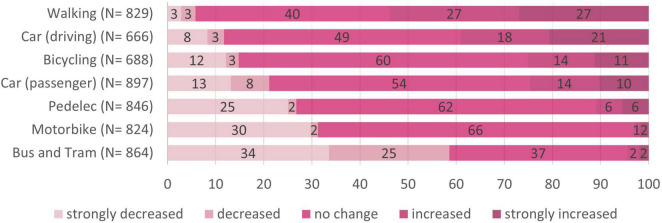
Transport mode shift from before to during the COVID-19 pandemic. *N* = 918; numbers on the bars represent percentages.

Transportation mode shift from before the pandemic to during the pandemic is illustrated in Figure 3. Most participants reported having strongly decreased or decreased their public transport use (i.e., bus and tram). In contrast to this, other modes of transport were increasingly used during the pandemic: 54% reported having increased or strongly increased walking, 39% report having increased or strongly increased car usage, and 25% reported having increased or strongly increased bicycling.

Socio-demographic characteristics for the four target groups can be found in Table 1. An explorative one-way ANOVA revealed significant differences concerning the age of participants in the four target groups, *F*(3, 914) = 5.35, *p* = 0.001. Follow-up pairwise comparisons (*p_crit_* = 0.008) revealed that loyal users (*M* = 43.99 years, *SD* = 21.46) were significantly younger as compared to non-users (*M* = 50.35 years, *SD* = 17.31), *p* = 0.003, *d* = 0.59, 95% CI [0.13; 0.52]. Chi squared tests (*p_crit_* = 0.008) also revealed large significant differences between the target groups concerning the distribution of occupational statuses, *d* = 0.60, 95% CI [0.47; 0.74], and participants’ dependency on public transport, *d* = 1.85, 95% CI [1.67; 2.02]. The results of follow-up pairwise comparisons can be found in Table 1. To sum them up, we found that ongoing users of public transport were less often employed and more often students than dropouts and non-users. They were more often dependent on public transport but also more often preferred public transport even though alternatives were present than dropouts and non-users. In turn, dropouts and non-users more often reported preferring alternatives over public transport or having no access to public transport.

### Infection Risk Perception in Public Transport

**FIGURE 4:**
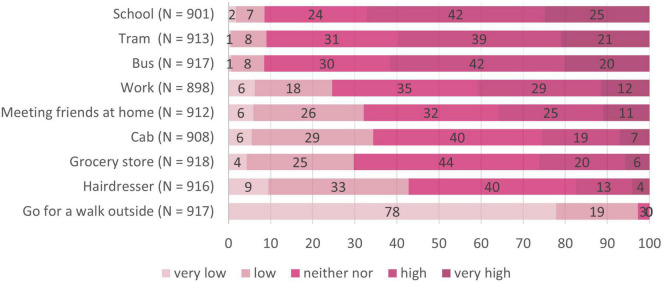
Subjective risk perception in different day-to-day situations. *N* = 918; numbers on the bars represent percentages.

The perceived infection risk for public transport in comparison with other day-to-day situations in the total sample is illustrated in Figure 4. A Wilcoxon signed rank test on paired samples revealed no difference concerning risk perception of using tram or bus (*p* = 0.307). We therefore did not differentiate between these two forms of public transport in the following tests and compared the other day-to-day situations with using the tram only. Multiple Wilcoxon signed rank test on paired samples (*p*_crit_ = 0.006) revealed that out of all situations considered, only the risk of attending school was evaluated significantly higher than the use of public transport (i.e., the tram), *V* = 56025, *p* = 0.004. All other situations, that is being in the workplace, *V* = 132533, *p* < 0.001, meeting friends at home, *V* = 171906, *p* < 0.001, taking a cab, *V* = 175925, *p* < 0.001, going to the grocery store, *V* = 158121, *p* < 0.001, going to the hairdresser, *V* = 225711, *p* < 0.001, and going for a walk, *V* = 396303, *p* < 0.001, were perceived as significantly less risky as compared to using the tram. We hence found public transport (i.e., bus and tram) to be ranked second highest after attending school.

**FIGURE 5:**
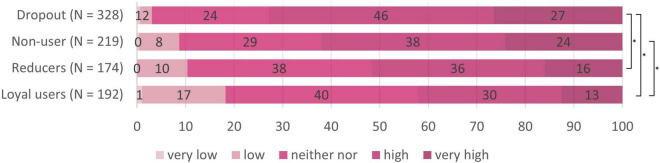
Subjective risk perception for using the tram in the different target groups. *N* = 918; numbers on the bars represent percentages. *p < 0.008 in the pairwise comparisons.

**FIGURE 6:**
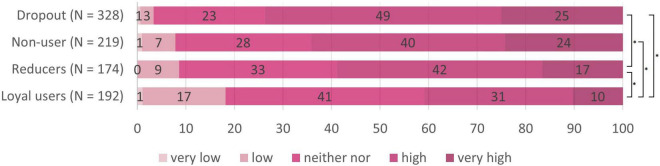
Subjective risk perception for using the bus in the different target groups. *N* = 918; numbers on the bars represent percentages. *p < 0.008 in the pairwise comparisons.

**FIGURE 7:**
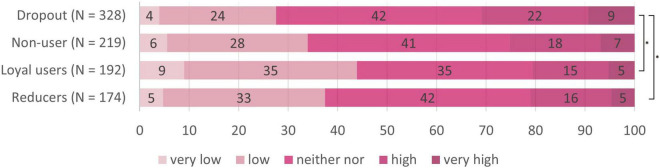
Subjective risk perception for using the cab in the different target groups. *N* = 918; numbers on the bars represent percentages. *p < 0.008 in the pairwise comparisons.

We now looked for differences concerning risk perception between the four target groups (see Table 2). Nine Kruskal Wallis tests (*p*_crit_ = 0.006) revealed differences between the target groups regarding perceptions of infection risk for using the tram, the bus, and the cab, but not for going to school, to work, meeting friends at home, going to a grocery store, going to the hairdresser, and going for a walk. Pairwise follow-up tests (*p*_crit_ = 0.008) revealed that loyal users perceived lower infection risk than reducers in buses, *W* = 20450, *p* < 0.001, *r* = 0.19, and trams, *W* = 18840, *p* < 0.001, *r* = 0.12, than dropouts in buses, *W* = 44123, *p* < 0.001, *r* = 0.34, trams, *W* = 42781, *p* < 0.001, *r* = 0.32, and cabs, *W* = 37354, *p* < 0.001, *r* = 0.17, and as non-users in buses, *W* = 27008, *p* < 0.001, *r* = 0.26, and trams, *W* = 26124, *p* < 0.001, *r* = 0.22 (see also the subscripts in Table 2). Hence, reducers, dropouts and non-users had an increased perception of infection risk for public transport compared to loyal users, that did however not generalize to other day-to-day situations (see also Figures 5–7) for the risk evaluations for using the tram, the bus and the cab.

**TABLE 2:** Infection risk as evaluated by the four target groups.

	Loyal (L) *n* = 193			Reducers (R) *n* = 175			Dropouts (D) *n* = 331			Non-users (N) *n* = 219			Total Sample *N* = 918				
	*n*	*Mdn*	*IQR*	*n*	*Mdn*	*IQR*	*n*	*Mdn*	*IQR*	*n*	*Mdn*	*IQR*	*N*	*Mdn*	*IQR*	*H*(3)	*p*
Bus*	193	3_R, D, N_	1	175	4_L, D_	1	331	4_L, R_	1.5	218	4_L_	1	917	4	1	61.92	<0.001
Tram*	192	3_D, N_	1	174	4_D_	1	328	4_L, R_	2	219	4_L_	1	913	4	1	58.08	<0.001
School	188	4	1	174	4	2	325	4	2	214	4	1	901	4	1	7.48	0.058
Meeting friends at home	193	3	2	171	3	2	330	3	2	218	3	2	912	3	2	1.53	0.676
Cab*	189	3_D_	1	171	3_D_	1	330	3_L, R_	2	218	3	1.75	908	3	2	17.96	<0.001
Grocery	193	3	2	175	3	1	331	3	2	219	3	2	918	3	2	2.35	0.504
Hairdresser	193	3	1	175	3	1	329	3	1	219	3	1	916	3	1	5.49	0.139
Work	185	3	1	174	3	1	323	3	1	216	3	2	898	3	1	7.28	0.063
Walk	192	1	1	175	1	0	331	1	0	219	1	0	917	1	0	6.85	0.077

Mdn, Median; IQR, interquartile range. *p < 0.003 (Bonferroni corrected for the 17 omnibus tests) in the Chi-squared omnibus test. Pairwise group comparisons are based on two-sided Pearson’s Chi-squared test with Yates’ continuity correction (Bonferroni corrected p_crit_ = 0.008). For each significant difference between the target groups, the letter of the significantly different group (L, loyal users; R, reducers; D, dropouts; N, non-users) appears as subscript in the column header and the other target group(s). Mdn: 5 = very high, 4 = high, 3 = neither nor, 2 = low, 1 = very low.

### Criteria of Transport Mode Selection

**FIGURE 8:**
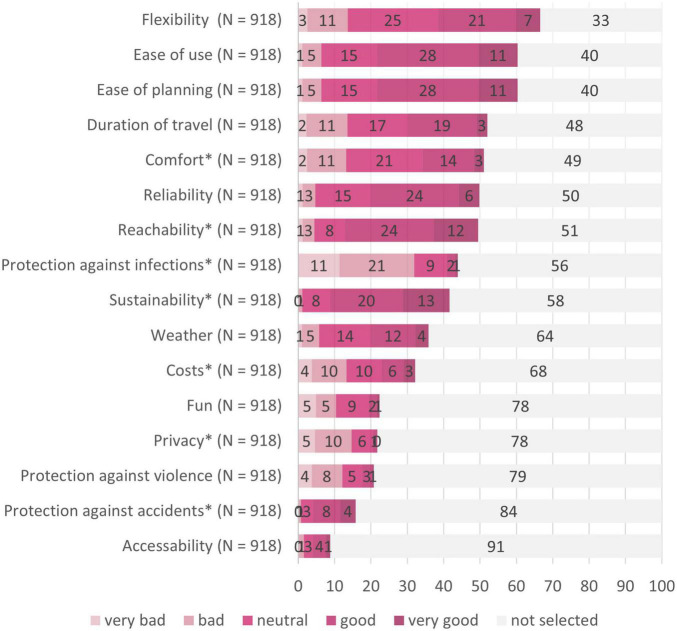
Evaluation of public transport in regards to the importance of different criteria. *N* = 918; Prot. scr., protective screens. Numbers on the bars represent percentages. *Indicates significant differences (*p* < 0.003) between the target groups in selecting this criterion.

Figure 8 summarizes the criteria determining transport mode choice during the pandemic in our sample. Flexibility, ease of use, planning, travel duration, and comfort were the top five selected criteria influencing transport mode selection.

Rankings of the criteria of transport mode selection in each of the four target groups can be found in the Supplementary (Supplementary Figures 1–4). In order to inspect differences in the distribution of the criteria between the target groups, we conducted fifteen Chi-squared tests (*p*_crit_ = 0.003; see Table 3). No differences between the groups were found concerning the importance of flexibility, ease of use, ease of panning, and duration of travel. These were the top criteria of transport mode selection in all four groups. However, differences between the target groups were found concerning the factors comfort, reachability, protection against infections, sustainability, costs, privacy, and protection against accidents.

**TABLE 3:** Criteria of transport mode choice as selected by the four target groups.

	Loyal (L) *n* = 193		Reducers (R) *n* = 175		Dropouts (D) *n* = 331		Non-users (N) *n* = 219		Total Sample *N* = 918			
	*n*	*%*	*n*	*%*	*n*	*%*	*n*	*%*	*n*	χ ^2^	*p*	*d*
Flexibility of use	114	59	107	61	236	71	154	70	630	11.97	0.007	
Ease of use	128	66	96	55	195	59	135	62	563	5.92	0.116	
Ease of planning	99	51	89	51	212	64	123	56	531	13.25	0.004	
Duration of travel	85	44	91	52	176	53	126	58	490	12.39	0.006	
Comfort*	86	45_N_	79	45_N_	180	54	124	57_L, R_	479	14.76	0.002	0.26
Reliability	90	47	100	57	171	52	97	44	467	6.83	0.078	
Reachability*	111	58_D, N_	105	60_D, N_	146	44_L, R_	92	42_L, R_	459	19.96	<0.001	0.30
Protection infections*	45	23_R, D_	79	45_L, D_	197	60_L, R, N_	82	37_D_	408	69.47	<0.001	0.57
Sustainability*	118	61_D, N_	99	57_D, N_	111	34_L, R_	54	25_L, R_	388	76.67	<0.001	0.60
Weather	67	35	67	38	118	36	77	35	341	0.30	0.961	
Costs*	85	44_D, N_	75	43_D, N_	84	25_L, R_	51	23_L, R_	299	35.20	<0.001	0.40
Fun	37	19	38	22	73	22	57	26	213	3.59	0.309	
Privacy*	14	7_D, N_	26	15_D, N_	89	27_L, R_	71	32_L, R_	206	52.37	<0.001	0.49
Protection violence	28	15	34	19	89	27	40	18	193	13.48	0.004	
Protection accidents*	45	23_D, N_	34	19	44	13_L_	22	10_L_	146	16.02	0.001	0.27
Lack of alternatives	27	14	27	15	28	8	28	13	110	6.74	0.081	
Accessibility	21	11	21	12	24	7	15	7	83	4.38	0.223	

*p < 0.003 (Bonferroni corrected for the 16 omnibus tests) in the omnibus Chi-squared test. Pairwise group comparisons are based on pairwise two-sided Pearson’s Chi-squared test with Yates’ continuity correction (Bonferroni corrected p_crit_ = 0.008). For each significant difference between the target group in the column header and the other target group(s), the letter of the significantly different group (L, loyal users; R, reducers; D, dropouts; N, non-users) appears as subscript.

We followed up these significant results by means of pairwise follow up Chi-squared tests (*p*_crit_ = 0.008). The results of these tests can be found as subscripts in Table 3. In the following, we will highlight the preferences of each target group in comparison to the other groups.

Loyal users evaluated sustainability, reachability, costs, and protection against accidents more often as important factor concerning their transport mode selection compared to one or more other groups. Sustainability was evaluated more often as important in comparison to dropouts, χ^2^(1, *N* = 524) = 34.97, *p* < 0.001, *d* = 0.40, 95% CI [0.27; 0.53] and non-users, χ^2^(1, *N* = 412) = 59.49, *p* < 0.001, *d* = 0.53, 95% CI [0.39; 0.66]. Reachability was evaluated significantly more often as important as compared to non-users, χ^2^(1, *N* = 412) = 12.78, *p* < 0.001, *d* = 0.24, 95% CI [0.11; 0.37] and dropouts, χ^2^(1, *N* = 524) = 8.51, *p* = 0.003, *d* = 0.19, 95% CI [0.06; 0.32]. Costs were evaluated more often as important as compared to dropouts, χ^2^(1, *N* = 524) = 18.92, *p* < 0.001, *d* = 0.29, 95% CI [0.16; 0.42], and non-users, χ^2^(1, *N* = 412) = 21.81, *p* < 0.001, *d* = 0.31, 95% CI [0.18; 0.44]. Protection against accidents was evaluated more often as important as compared to dropouts, χ^2^(1, *N* = 524) = 7.99, *p* = 0.005, *d* = 0.19, 95% CI [0.06; 0.32] and non-users, χ^2^(1, *N* = 412) = 10.74, *p* = 0.001, *d* = 0.22, 95% CI [0.09; 0.35]. To conclude, loyal users were not as concerned about infection risk as other groups were, and one of the most important criteria for loyal users for keeping on using public transport was sustainability, but also reachability and costs.

Reducers evaluated reachability, sustainability, protection against infections and costs significantly more often as important criteria for transport mode selection as compared to other groups. Protection against infection was evaluated more often as important as compared to loyal users, χ^2^(1, *N* = 368) = 17.64, *p* < 0.001, *d* = 0.28, 95% CI [0.15; 0.41]. Reachability evaluated more often as important as compared to dropouts, χ^2^(1, *N* = 506) = 10.51, *p* = 0.001, *d* = 0.22, 95% CI [0.09; 0.35], and to non-users, χ^2^(1, *N* = 394) = 10.10, *p* = 0.001, *d* = 0.21, 95% CI [0.08; 0.34]. Sustainability was evaluated more often as important as compared to dropouts, χ^2^(1, *N* = 506) = 23.91, *p* < 0.001, *d* = 0.33, 95% CI [0.20; 0.46], and non-users, χ^2^(1, *N* = 394) = 38.00, *p* < 0.001, *d* = 0.42, 95% CI [0.28; 0.55]. Costs were evaluated more often as important as compared to dropouts, χ^2^(1, *N* = 506) = 14.83, *p* < 0.001, *d* = 0.26, 95% CI [0.13; 0.39] and non-users, χ^2^(1, *N* = 394) = 14.59, *p* < 0.001, *d* = 0.25, 95% CI [0.12; 0.38]. To conclude, in contrast to loyal users, reducers considered infection as a relevant criterion for transport mode selection. However, this group also considered sustainability, reachability and costs as important criteria for the continued use of public transport during the pandemic.

Dropouts evaluated protection against infections and privacy significantly more often as important criteria for transport mode selection as compared to other groups. Protection against infections was evaluated more often as important as compared to loyal users, χ^2^(1, *N* = 524) = 63.03, *p* < 0.001, *d* = 0.54, 95% CI [0.41; 0.68], reducers, χ^2^(1, *N* = 506) = 9.78, *p* = 0.001, *d* = 0.21, 95% CI [0.08; 0.34], and non-users, χ^2^(1, *N* = 550) = 24.13, *p* < 0.001, *d* = 0.33, 95% CI [0.20; 0.46]. Privacy was evaluated more often as important as compared to loyal users, χ^2^(1, *N* = 524) = 31.32, *p* < 0.001, *d* = 0.38, 95% CI [0.24; 0.51] and reducers, χ^2^(1, *N* = 506) = 10.43, *p* = 0.001, *d* = 0.38, 95% CI [0.24; 0.51]. To conclude, infection risk was a highly relevant criterion for dropouts to change to other modes of transportation than public transport.

Finally, non-users evaluated comfort and privacy significantly more often as a criterion for transport mode selection as compared to other groups. Comfort was evaluated more often as important as compared to loyal users, χ^2^(1, *N* = 412) = 12.47, *p* < 0.001, *d* = 0.23, 95% CI [0.10; 0.37] and reducers, χ^2^(1, *N* = 394) = 8.36, *p* < 0.004, *d* = 0.19, 95% CI [0.06; 0.32]. Privacy was evaluated more often as important as compared to loyal users, χ^2^(1, *N* = 412) = 43.14, *p* < 0.001, *d* = 0.44, 95% CI [0.31; 0.58] and reducers, χ^2^(1, *N* = 394) = 16.68, *p* < 0.001, *d* = 0.27, 95% CI [0.14; 0.40]. To conclude, non-users did not consider the pandemic as a very relevant issue in their selection of transport mode but focused stronger on aspects of comfort, convenience, and privacy.

### Satisfaction With the Criteria of Transport Mode Selection in Public Transport

In addition to the frequency of selected criteria, participants’ satisfaction with these criteria in public transport are illustrated in Figure 8. The satisfaction of four of the top five criteria of transport mode selection (i.e., flexibility, planning, travel duration, and comfort) by public transport was evaluated as neither good nor bad in the total sample. Ease of use as further top criterion was evaluated as well fulfilled. Public transport received the best five evaluations for ease of use, reliability, reachability, sustainability, protection against accidents, and accessibility; all five factors were evaluated as good. The least well evaluated factors concerning public transport were protection against infections, privacy, and protection against violence, all three were evaluated as bad.

Detailed evaluations of the fulfilment of criteria in public transport, displayed separately for the four target groups, can be found in Table 4 and is also illustrated in the Supplementary (Supplementary Figures 1–4). We looked for differences between the target groups by comparing the medians with 15 Kruskal Wallis tests (*p*_crit_ = 0.003). The results of these omnibus tests and the descriptive statistics can be found in Table 4 as well. The tests revealed significant differences between one or more groups for all criteria besides accessibility, privacy, and protection against accidences: All four target groups agreed that accessibility and protection against accidents were rather well fulfilled in public transport, whereas privacy was not given.

**TABLE 4:** Evaluation of criteria regarding public transport evaluated by the four target groups.

	Loyal (L) *n* = 193			Reducers (R) *n* = 175			Dropouts (D) *n* = 331			Non-users (N) *n* = 219			Total Sample *N* = 918				
	*n*	*Mdn*	*IQR*	*n*	*Mdn*	*IQR*	*n*	*Mdn*	*IQR*	*n*	*Mdn*	*IQR*	*N*	*Mdn*	*IQR*	*H*	*p*
Flexibility of use*	114	4_D, N_	2	107	4_D, N_	1	236	3_L, R_	1	154	3_L, R_	1	611	3	1	90.57	<0.001
Ease of use*	128	4_D, N_	1	96	4_D, N_	1	195	4_L, R_	1	135	3_L, R_	1	554	4	1	106.53	<0.001
Ease of planning*	99	4_D, N_	1	89	4_D, N_	1	212	3_L, R_	2	123	3_L, R_	2	523	3	1	97.22	<0.001
Duration of travel*	85	4_D, N_	1	91	4_D, N_	1	176	3_L, R_	2	126	3_L, R_	2	478	3	2	70.99	<0.001
Comfort*	86	4_D, N_	1	79	4_D, N_	1	180	3_L, R_	1	124	3_L, R_	2	469	3	2	61.07	<0.001
Reliability*	90	4_D, N_	0.75	100	4_D, N_	1	171	3_L, R_	1	97	3_L, R_	1	458	4	1	37.70	<0.001
Reachability*	111	4_D, N_	1	105	4_D, N_	1	146	4_L, R_	1	92	4_L, R_	1	454	4	1	48.32	<0.001
Protection infections*	45_D_	3_R, D, N_	1	79	2_L_	1	197	2_L_	1	82	2_L_	2	403	2	2	28.25	<0.001
Sustainability*	118	4_D, N_	1	99	4_N_	1	111	4_L_	0	54	4_L, R_	1	382	4	1	26.57	<0.001
Weather*	67	4_D, N_	1	67	4_D, N_	1	118	3_L, R, N_	1	77	3_L, R, D_	1	329	3	1	56.70	<0.001
Costs*	85	3_D, N_	2	75	3_D, N_	2	84	3_L, R_	1	51	2_L, R_	1	295	3	2	26.89	<0.001
Fun*	37	2_D, N_	2	38	3_N_	1	73	2_L_	1	57	2_L R_	2	205	3	1	21.19	<0.001
Privacy	14	2.5	1	26	2	1	89	2	1	71	2	1	200	2	1	3.271	0.352
Protection violence*	28	3_N_	1.25	34	3_D, N_	2	89	2_R_	1	40	2_L, R_	1.25	191	2	1	27.52	<0.001
Protection accidents	45	4	2	34	4	1	44	4	1	22	4	1	145	4	2	4.63	0.201
Accessibility	21	4	1	21	4	1	24	3.5	1	15	3	1.5	81	4	1	6.59	0.086

Mdn, Median; IQR, interquartile range. *p < 0.003 (Bonferroni corrected for the 16 omnibus tests) in the omnibus Kruskal Wallis test. Pairwise group comparisons are based on two-sided Wilcoxon tests (Bonferroni corrected p_crit_ = 0.008). For each significant difference between the target group in the column header and the other target group(s), the letter of the significantly different group (L, loyal users; R, reducers; D, dropouts; N, non-users) appears as subscript. Mdn: 5 = very good, 4 = good, 3 = neither nor, 2 = bad, 1 = very bad.

We followed up the significant results with pairwise two-sided Wilcoxon tests (*p_crit_* = 0.008). The results of these tests can be found as subscripts in Table 4. In the following sections we highlight significant differences between the target groups concerning their perceived deficits of public transport.

Loyal users evaluated all criteria either as well fulfilled in public transport as other target groups, or better. Hence, they had a more positive attitude about public transport than all other groups.

Reducers evaluated protection against infections as significantly less well fulfilled in public transport as compared to loyal users, *W* = 1286, *p* = 0.007, *r* = 0.24. All other criteria were evaluated either on the same level fulfilled in public transport, as by other target groups, or better. Hence, reducers too, had a more positive attitude about public transport than those who did not use public transport during the pandemic, but in contrast to loyal users they perceived more deficits in regards to protection against infections in public transport.

Dropouts evaluated flexibility, ease of planning, duration of travel, comfort, reliability, protection against infections, weather, protection against violence and accessibility as less well fulfilled in public transport than other groups: Flexibility of use was evaluated less well as compared to loyal users, *W* = 7636, *p* < 0.001, *r* = 0.37, and to reducers, *W* = 8687, *p* < 0.001, *r* = 0.26. Ease of planning was evaluated by dropouts less well as compared to loyal users, *W* = 5064, *p* < 0.001, *r* = 044, and to reducers, *W* = 5965, *p* < 0.001, *r* = 0.30. Duration of travel was evaluated less well as compared to loyal users, *W* = 4108, *p* < 0.001, *r* = 0.39, and to reducers, *W* = 4618, *p* < 0.001, *r* = 0.36. Comfort was evaluated less well as compared to as compared to loyal users, *W* = 4260, *p* < 0.001, *r* = 0.38, and to reducers, *W* = 3983, *p* < 0.001, *r* = 0.37. Reliability was evaluated less well as compared to loyal users, *W* = 5062, *p* < 0.001, *r* = 0.30 and to reducers, *W* = 6488, *p* < 0.001, *r* = 0.22. Protection against infections was evaluated less well as by loyal users, *W* = 2418, *p* < 0.001, *r* = 0.33. Weather was evaluated less well as compared to loyal users, *W* = 2532, *p* < 0.001, *r* = 0.32, and to reducers, *W* = 2032, *p* < 0.001, *r* = 0.43. Protection against violence was evaluated worse as compared to reducers, *W* = 869, *p* < 0.001, *r* = 0.34. To sum up: Like reducers, participants who had dropped out of public transport during the pandemic perceived protection against infections not only more often as important, but also as less well fulfilled in public transport. In addition, they perceived more deficits than ongoing users, for instance regarding their other most preferred criteria for transport mode selection (i.e., flexibility, ease of planning, travel duration and comfort).

Finally, non-users evaluated flexibility, ease of use, ease of planning, duration of travel, comfort, reliability, protection against infections, weather, costs, protection against violence and accessibility significantly less well than other groups: Flexibility of use was evaluated less well as by loyal users, *W* = 4014, *p* < 0.001, *r* = 0.48, and by reducers, *W* = 4014, *p* < 0.001, *r* = 0.14. Ease of planning was evaluated less well than by loyal users, *W* = 2375, *p* < 0.001, *r* = 0.55, and reducers, *W* = 2928, *p* < 0.001, *r* = 0.16. Duration of travel was evaluated less well as by loyal users, *W* = 2908, *p* < 0.001, *r* = 0.41, and reducers, *W* = 3260, *p* < 0.001, *r* = 0.39. Comfort was evaluated less well as by loyal users, *W* = 3432, *p* < 0.001, *r* = 0.32, and reducers, *W* = 3247, *p* < 0.001, *r* = 0.30. Reliability was evaluated less well as by loyal users, *W* = 2729, *p* < 0.001, *r* = 0.35, and reducers, *W* = 3522, *p* < 0.001, *r* = 0.26. Protection against infections was evaluated less well as by loyal users, *W* = 1182, *p* = 0.004, *r* = 0.31. Weather was evaluated less well as by loyal users, *W* = 1464, *p* < 0.001, *r* = 0.39, and reducers, *W* = 1140, *p* < 0.001, *r* = 0.51. Protection against violence was evaluated less well as by loyal users, *W* = 297, *p* = 0.006, *r* = 0.41, and reducers, *W* = 295, *p* < 0.001, *r* = 0.50. To sum it up, non-users perceived more deficits in public transport as compared to ongoing users (i.e., loyal and reducers) in terms of their most important criteria flexibility, ease of use and planning, travel duration and comfort, but also for infection risk which was however not that important for transport mode selection in this group.

### Evaluation of Measures Taken in Public Transport to Decrease the Infection Risk

**FIGURE 9:**
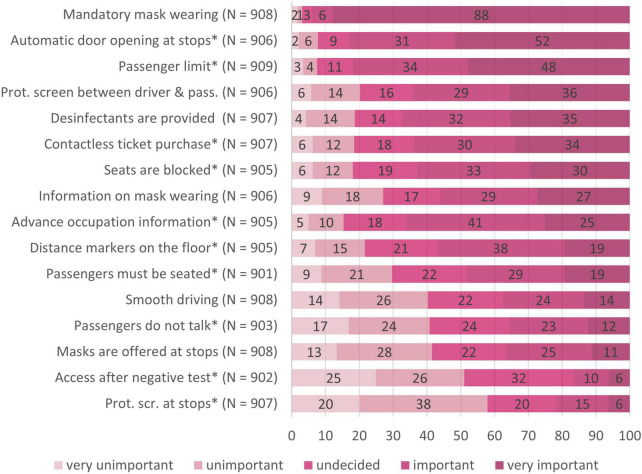
Evaluation of the different measures taken against COVID-19 in public transport. *N* = 918; numbers on the bars represent percentages. *Indicates that significant differences (*p* < 0.003) were found between the target groups in evaluating this measure.

Figure 9 gives an overview of the participants’ evaluations of the fifteen measures taken against COVID-19 transmission in public transport. The top ranked measures were wearing the mask and doors open automatically at stops, being both evaluated as very important. Participants evaluated as important that one seat is blocked in between, disinfectants are provided, information about mask wearing is provided, passenger limit, distance markers at stops, screens between drivers and passengers, contactless ticket purchase and access to advanced occupation information. The measures evaluated as unimportant were access to public transport only with negative test and protective screens at the stops. In summary, measures that maintained social distance and fresh air in public transport were rated as particularly important by the sample.

A summary of the four target groups concerning their evaluations of sixteen measures against COVID-19 transmission in public transport can be found in Table 5 and is furthermore illustrated in the Supplementary (Supplementary Figures 5–8). Kruskal Wallis tests (*p*_crit_ = 0.003) (see Table 5) revealed no differences between the four target groups concerning the evaluation of mandatory mask wearing, providing disinfectants, information about masks, screen between driver and passenger, masks can be purchased, and smooth driving. However, significant differences between the target groups were found in the evaluations of automatic door opening, seats blocked, passenger limit, distance markers, contactless ticket purchase, occupation information, passengers must be seated, passengers do not talk, access only after test and protective screens at stops. We followed up the significant results with pairwise two-sided Wilcoxon tests (*p_crit_* = 0.008). The results of these tests can be found as subscripts in Table 5. In the following sections we highlight significant differences of preferences between the target groups:

**TABLE 5:** Evaluation of the different measures taken against COVID-19 in public transport by the four target groups.

	Loyal *n* = 193			Reducers *n* = 175			Dropouts *n* = 331			Non-users *n* = 219			Total Sample *N* = 918				
	*n*	*Mdn*	*IQR*	*n*	*Mdn*	*IQR*	*n*	*Mdn*	*IQR*	*n*	*Mdn*	*IQR*	*N*	*Mdn*	*IQR*	*H*	*p*
Mandatory mask wearing	192	5	0	173	5	0	328	5	0	215	5	0	901	5	0	4.95	0.175
Doors open automatically*	191	5_R_	1	172	5_L, D, N_	1	327	4_R_	1	216	4_R_	2	906	5	1	30.12	<0.001
Seats blocked*	189	3_R, D, N_	2	172	4_L, D_	2	329	4_L, R_	2	215	4_L_	2	905	4	2	50.56	<0.001
Disinfectants are provided	190	4	2	174	4	2	327	4	2	216	4	2	907	4	2	4.21	0.240
Mask information provided	190	4	3	173	4	2	328	4	3	215	4	2	909	4	3	2.79	0.424
Passenger limit*	190	4_R, D, N_	2	175	4_L, D_	1	329	5_L, R_	1	215	4_L_	1	905	4	1	50.87	<0.001
Distance markers at stops*	191	3_D_	2	173	4	1	326	4_L_	1	215	4	1	908	4	1	25.55	<0.001
Screen between driver and pass.	191	4	2	174	4	2	327	4	2	214	4	3	906	4	2	6.65	0.084
Contactless ticket purchase*	190	4_D_	2	174	4_D_	2	327	4_L,R,N_	2	216	4_D_	2	907	4	2	19.77	<0.001
Occupation information*	190	4_D_	1	175	4_D_	2	327	4_L, R_	2	213	4	1	905	4	2	19.66	<0.001
Masks can be purchased	191	3	2	173	3	2	328	3	2	216	3	2	908	3	2	0.51	0.917
Passengers must be seated*	191	3_D, N_	2	173	3_D, N_	2	323	4_L, R_	1	214	4_L, R_	1.75	901	3	2	31.19	<0.001
Smooth driving	190	3	2	175	3	2	328	3	2	215	3	2	908	3	2	2.89	0.409
Passengers do not talk*	190	2.5_D_	2.75	174	3_D_	2	325	3_L, R_	2	214	3	2	903	3	2	16.27	<0.001
Access only after test*	190	2_D, N_	2	173	2	2	325	3_L_	1	214	2.5_L_	1	902	2	1	32.58	<0.001
Protective screens at stops*	191	2_D_	2	174	2_D_	1	327	2_L, R_	1.5	215	2	1	907	2	1	17.30	<0.001

Mdn, Median; IQR, interquartile range. *p < 0.003 in the omnibus Kruskal Wallis test (Bonferroni corrected for the 16 omnibus tests). Pairwise group comparisons are based on two-sided Wilcoxon tests (Bonferroni corrected p_crit_ = 0.008). For each significant difference between the target groups, the letter of the significantly different group (L, loyal users; R, reducers; D, dropouts; N, non-users) appears as subscript under the median. For each significant difference between the target group in the column header and the other target group(s), the letter of the significantly different group (L, loyal users; R, reducers; D, dropouts; N, non-users) appears as subscript. Mdn: 5 = very important, 4 = important, 3 = neither nor, 2 = unimportant, 1 = very unimportant.

Loyal users did not evaluate any measure against COVID-19 transmission in public transport as significantly more important that other groups.

Reducers, in contrast, preferred automatic door opening and blocked seats significantly more than other target groups: Automatic door opening was preferred more than by loyal users, *W* = 19044, *p* = 0.003, *r* = 0.16, than by dropouts, *W* = 22888, *p* < 0.001, *r* = 0.17, and non-users, *W* = 13208, *p* < 0.001, *r* = 0.27. Blocked seats were perceived as more important than by loyal users, *W* = 19080, *p* < 0.001, *r* = 0.15. In sum, reducers evaluated measures that were directly experienceable within the vehicle as significantly more important than other groups.

Dropouts evaluated blocked seats, a passenger limit, distance markers at stops, passengers must be seated, passengers do not talk and entrance after test significantly more important than other groups: Blocked seats were evaluated as more important than by loyal users, *W* = 42054, *p* < 0.001, *r* = 0.31. A passenger limit was evaluated as more important than by loyal users, *W* = 42016, *p* < 0.001, *r* = 0.31 and by reducers, *W* = 32682, *p* < 0.001, *r* = 0.12. Distance markers at stops were evaluated as more important than by loyal users, *W* = 42016, *p* < 0.001, *r* = 0.22. Passengers must be seated was evaluated as more important than by loyal users, *W* = 38290, *p* < 0.001, *r* = 0.21, and reducers, *W* = 33034, *p* = 0.005, *r* = 0.16. Passengers do not talk was evaluated as more important than by loyal users, *W* = 39778, *p* < 0.001, *r* = 0.21. Entrance after negative test was evaluated as more important than by loyal users, *W* = 39778, *p* < 0.001, *r* = 0.25. In sum, dropouts perceived most measures against the pandemic as important and differed from other groups especially regarding their evaluation of a passenger limit as very important and of distance markers at stops as important. Hence concerns about crowding seemed to be a very important criterion for dropping out of public transport during the pandemic.

Non-users evaluated blocked seats, passengers must be seated, and access after test, as significantly more important compared to other groups: Blocked seats were perceived as more important than by loyal users, *W* = 25374, *p* < 0.001, *r* = 0.22. Passengers must be seated was evaluated as more important than by loyal users, *W* = 25173, *p* < 0.001, *r* = 0.21. Access after test was evaluated as more important as compared to loyal users, *W* = 24201, *p* = 0.006, *r* = 0.17. In sum, non-users also perceived many measures against the pandemic as important. They differed from other groups concerning their positive evaluation of access after tests and mandatory seating in vehicles.

## Discussion

In this study, we aimed to understand passenger reductions in public transport during the pandemic and sought leverage to bring people (back) to public transport after the pandemic. We initially quantified changes in public transport use during the pandemic in a representative sample of a German major city. Within this sample, we found differences between the target groups of pandemic public transport usage (i.e., loyal users, reducers, dropouts and non-users) regarding (1) their perceptions of infection risk, (2) their general preferences for mobility and these preferences’ fulfilment in public transport, and (3) their evaluations of measures taken by public transport suppliers against the pandemic.

In line with other studies (Finbom et al., 2020; Shibayama et al., 2021; DLR Transport, 2022a,b), we observed in our representative sample a general mode shift from public transport to walking, car usage, and bicycling during the pandemic. In our sample, only 21% kept on using public transport, 19% reduced public transport usage during the pandemic, 36% dropped out of public transport, and 24% remained general non-users of public transport.

Overall, we found participants to perceive a high infection risk in public transport. This increased perception of infection risk stands in contrast to other considerations (Sommer et al., 2021) that the objective infection risk is rather low in public transport, considering the short contact duration, the effective fresh air ventilation systems in and the fact that mask wearing has become the new norm. Participants hence tended to overestimate the infection risk for public transport in comparison to other situations. Looking at the different target groups, reducers, dropouts, and non-users perceived an increased (i.e., high) infection risk for public transport in comparison to loyal users. It is important to note that these differences in risk perception between groups were specific to public transport and did not generalize to comparable day-to-day situations, such as going to work, or to the grocery store. These findings are in line with Finbom et al. (2020), who found lower risk perception in users as compared to non-users, and with Sadique et al. (2007) who reported that even though a person’s perceived risk for influenza had little effect on their everyday lives, it did affect public transport usage. These findings allow for two possible explanations. First, an increased infection risk perception, specific to public transport might have caused reductions and dropouts of public transport during the pandemic. Second, people who stuck to public transport (because they had to do so, or because they wanted to) had the opportunity to experience that measures against the pandemic were also taken in public transport, that public transport was quite empty during the pandemic, or that, even though they used public transportation, they were not infected more often than others. These experiences might have resulted in a lower infection risk perception in the group of ongoing users. Both explanations, however, imply that decreasing the objective and subjective infection risk in public transport should be prioritized in order to (re-)attract passengers during the ongoing pandemic and in the future.

Looking at general criteria of transport mode selection during the pandemic, flexibility, ease of use, ease of planning, and travel duration were the top criteria for transport mode selection in all target groups. Loyal users however distinguished from other groups by a stronger consideration of sustainability, reachability, and costs. In the same vein, reducers selected their transport mode more often based on sustainability, reachability, and costs, but they rated (an insufficient) protection against infections as important, too. For dropouts this factor of (an insufficient) protection against infections was particularly often relevant, in addition to privacy. In contrast to that, non-users showed a stronger preference of comfort and privacy as compared to all other groups. Infection risk was thereby not very informative of their transport mode selection. Furthermore, participants in all four target groups agreed that reliability, reachability, and sustainability were well fulfilled by public transport, and that privacy was not fulfilled. A central finding in our representative sample was that reducers as well as dropouts and non-users perceived more deficits regarding protection against infections than loyal users. To sum it up, especially in the group of reducers and dropouts, protection against infection was a relevant criterion to reduce or quit public transport usage during the pandemic, especially as this factor was perceived as not well fulfilled in public transport by these groups.

Looking at participants’ evaluation of the measures taken in public transport against the pandemic, mandatory mask wearing was evaluated as the topmost important measure to feel safe in public transport during the pandemic in all four groups. In addition, reducers regarded automatic door opening very important and thereby more important than all other groups. Dropouts perceived a passenger limit as very important, and regarded blocked seats, distance markers as stops, mandatory seating, and access with negative test as more important measures than other groups. Non-users perceived blocked seats, mandatory seating, and access with negative test as more important measures than other groups.

Finally, we found ongoing public transport users (i.e., loyal users and reducers) to be on average younger, more often students, being more likely to have no access to alternatives to public transport, or simply preferring public transport as compared to non-users. Non-users (i.e., pandemic dropouts and non-users) were on average older, more often employed, had more often access to alternatives to public transport or no access to public transport. The differences in socio-demographic support the findings of Finbom et al. (2020) that context factors, such as dependency on public transport, access to public transport, occupational status, as well as participants age (and accordingly their objective risk to suffer severely from a COVID-19) determined transport mode selection during the pandemic as well, and that these factors should be considered when planning to (re-)attract these groups. Contrarily to Finbom et al. (2020), we did not find any differences in the financial statuses of non-users and users.

### Practical Implications for Communication in Public Transport

Since the beginning of the pandemic, there have been intensive discussions about how to win back passengers and make public transport more resistant to pandemics. In the following section we will discuss communicative interventions to decrease the objective and subjective risk in public transport during the pandemic with a focus on the four target groups described in this paper. Our thoughts are based on the summary of behavioral change techniques of Contzen and Mosler (2015).

#### Using Communication to Decrease the Objective Risk in Public Transport

In the dynamic setting of the pandemic, targeted communication can play an important role in communicating the state-of-the-art knowledge about the most important measures, and to guide desired behavior in passengers of public transport. In the beginning of the COVID-19 pandemic, when it was yet unclear how the virus was transmitted, prompting the population to stay at home was an important communicative measure to decrease the objective risk in public transport, in particular because following the recommendation of keeping 1.5 m distance could only be fulfilled when occupation was low (McKinsey and Company, 2020). At the current moment, findings suggest that masks – especially FFP2 masks – are a very effective tool for decreasing infection susceptibility, even in crowded public transport (Chu et al., 2020; Ueki et al., 2020; Bagheri et al., 2021; Will et al., 2021). As long as recommendations concerning mask wearing, social distance and hygiene measures are followed by all passengers of a public transport, passengers thus could travel safely.

Loyal users and reducers are the main target groups, for communication addressing compliance with hygiene regulations within public transport. Even before entering public transport, they should be informed about digital measures of social distancing, such as the occupation tool and the option of contactless ticket purchase. As these instruments were newly implemented in public transport in many cities, customers need to be informed about the existence of these new options and first attempts in using these tools could be supported by instructional aids, or demonstrations. Trying out these tools could be prompted in a planning app, but other means of mass communication might be valuable tools of communication additionally. At stops and within the vehicle, nudges (e.g., distance markers on the floor) can be used to avoid crowding. Information about the correct do’s and don’ts in public transport should be communicated in a clearly visible fashion in the vehicles, but also outside of the vehicles to allow passengers to take the necessary preparations before entering (e.g., putting on the mask). Our findings show that all groups considered mandatory mask wearing as very important measure to feel safe in public transport. Prompting to wear the masks might thus seem a bit like preaching to the converted, but this measure is still important in order to establish correct mask wearing as the new norm and to prevent social loafing (Simms and Nichols, 2014). Passengers could thereby be informed of the increased protection of FFP2 masks in comparison to other medical masks. In addition, personal norms and self-efficacy belief could be activated. Thereby, loyal users (who did not perceive an increased risk in public transport) might profit more from a message focusing on altruistic values (e.g., “I wear the mask to protect others,” or “for others to feel safe”), or messages communicating mask wearing as a precondition to help public transport to keep on fulfilling its role as sustainable infrastructure even through the pandemic (e.g., “I am green! From mask to transport mode,” “I wear the mask to keep public transport working”). In contrast, reducers might be more receptive to a message highlighting their self-efficacy (e.g., “with the FFP2 mask I am safe”). When thinking about the new norm in public transport, it should also be considered that dropouts and non-users (re)entering public transport after a long time, or even for the first time, need to be informed about the altered regulations and the new normal of public transport in advance, to avoid drawbacks caused by a feeling of unfamiliarity or unpreparedness. Offering masks at the stations might be especially valuable for these groups. Furthermore, the study of Huu Manh et al. (2021) suggests that communication does not end with only wearing the mask, but also prompting to wear masks correctly could contribute to decreasing the objective risk in public transport. In their study, 11% of passengers failed to wear the mask correctly, especially passengers who were older, rarely used public transport, transported heavy luggage, or traveled with others. The study of Bagheri et al. (2021) highlights the additional value of wearing the mask correctly and the knowledge of how to do so should thus be communicated at the entrance of vehicles as well. In the same vein, providing disinfectant could be accompanied by prompts to use it and how to use it correctly. As reducers and dropouts in our study reported higher preference of measures that increased distance, it might also be helpful to find out which spaces in vehicles are the safest and to communicate these areas during a pandemic (or restrict access to risk groups).

#### Decreasing the Subjective Risk in Public Transport

Our findings show that public transport urgently needs to regain trust and the reputation of being a safe space after 2 years of the pandemic. Participants in our sample – especially reducers, dropouts and non-users – had an increased perception of infection risk that was specific to public transport. An important pre-condition for regaining public trust is of course to know about the objective infection risk in public transport and implementing the most effective measures to decrease it. However, technical solutions *per se* will not be enough. Insights from aerosol simulations that were conducted within our research project indicate that the filtration systems in public transport vehicles are very efficient, even though not directly experienceable for passengers (EMILIA Findings with ESI Group, 2021). Such findings on the objective infection risk should be communicated publicly, to adjust public perceptions of public transport. It is interesting to note that, according to our simulations, additional automatic door opening – though strongly preferred by ongoing users of public transport in our sample – only have a marginal additional effect (max. 10%) as compared to ventilation only and for that reason have more value as an instrument for decreasing the subjective risk perception (EMILIA Findings with ESI Group, 2021). This example shows that the most important objective measures are not always perceived accurately by passengers. Targeted communication might play an important role in adjusting risk perceptions and the evaluation of certain measures.

Loyal users and reducers could thereby potentially take on the important roles of knowledge multipliers for other target groups. Measures taken should therefore be highlighted and, where necessary, explained to passengers. Fact sheets on infection risks in relation to different measures/scenarios could support communication, as well as simulations, e.g., illustrating air exchange within the vehicle, might play an important role in showing the unseen measures taken in public transport and to increase self-efficacy. As we have found that loyal users and reducers perceive automatic door opening as a very important measure (even though the efficacy of this measure is limited according to scientific evidence), the underlying concern of getting fresh air into the vehicle can be taken as an opportunity to communicate the effectiveness of the ventilation system, which already brings fresh air into the vehicle (i.e., “you do not have to freeze for fresh air”). Once users of public transport have noticed which measures are taken in public transport and why they are taken, they can be prompted to speak with others about these measures to increase descriptive norms of using public transport within the pandemic. A creative way of transporting new behavioral norms of public transport to other groups, might be for instance to distributing masks as incentives and starting a photo competition with these masks in public transports. Within the vehicle one should keep in mind to only communicate measures that are possible to fulfill (e.g., unlike the often-seen recommendation of hand washing, which is simply not fulfillable in busses and trams) to maintain self-efficacy. In order to create a new image of public transport as a safe and hygienic space it might also be useful to perform hygiene measures that are very visible and experienceable for passengers (e.g., cleaning the surfaces, opening the doors automatically) as a mean of communication in itself.

The most challenging task will however be regaining the trust of pandemic dropouts. This group might have already established new habits and has had no opportunity to perceive the measures taken during the pandemic since it has fully avoided public transport. An important precondition to reattracting this group will be to create a new image of public transport. Public transport should transparently communicate about and reattribute past failure (“We were not prepared – now we are!”) and actively communicate what has been learned during the pandemic and which measures were taken to increase safety from infections and how they can be used (e.g., the occupation tool, new behavior rules). To disrupt the new habits of pandemic dropouts and creating opportunities to make new experiences in public transport, special discount campaigns (e.g., a ticket for free to experience the “new hygienic” public transport) might be useful to entice this group. However, these incentives should be guided by communication on measures against infection risk in public transport (e.g., with factsheets about the effectiveness of masks). A good idea might also be the active signaling of personal values, such as “We care about your health.” A very creative idea to signal personal values while creating opportunities for new experiences in public transport was for instance to invite passengers for a free ride to their vaccination (Steeger, 2021). Former subscribers could be contacted *via* letter, and other dropouts could be reached *via* mass media and *via* outdoor advertising, e.g., on the surface of the vehicles (e.g., stickers, highlighting the well ventilation inside). It should however be taken care that a positive group identity can be established in this group (e.g., “We are the comebacks of 2022!”, or “Boostered and back on the road”). It might also be important to anticipate and prompt coping with relapses and barriers when coming back (“If you feel unsafe, contact us!”, or “If you feel unsafe, wear your FFP2 mask – you will be safe!”).

Non-users could be attracted with conventional means of communication and incentives. They were not so much concerned about protection against infections, but rather concerned about comfort in public transport. This aspect should certainly be a part of communication when attracting this group, such as highlighting how convenient it is to drive to the city center without needing to search for a parking spot. In addition, attitudes regarding other factors such as flexibility, ease of use and planning, and travel duration could be altered. Distributing “A beginners guide for public transport after COVID-19,” including advice on behavior and hygiene but also showing example routes, comparing travel durations for bus and car including the search for parking spots, and providing how-to knowledge on public transport usage, ticket purchases and so on could be a successful mean of communication. In addition, incentives of free rides should also be combined with information on how to comfortably use the ticket, e.g., when linked with a specific event, an example route could be already planned out. Also, for the group of non-users, barrier planning, and coping should be taken into consideration by providing information on where to look if a vehicle is too late, where to enter to find a free seat, etc.

Taken altogether, a great variety of tailored measures are conceivable in order to bring people (back) to public transport, measures that take the specific characteristics of the different target groups into account, as derived from our study.

### Strengths and Limitations

In this study, we investigated public transport usage during the pandemic through a multifaceted lens. In contrast to other studies in the field, we managed to shed a light on the perspective of pandemic dropouts and non-users as well by recruiting a representative sample of a German major city. Our findings focus on risk perception during the pandemic, but we also go one step further in seeking and finding leverage points to (re-)attract passengers *via* targeted communication and to make public transport resilient to upcoming pandemics. Our findings are limited however by the focus on an urban setting and our findings should always be interpreted in context of the third wave and the second lockdown (Kodzo and Imöhl, 2022) in which we conducted our sampling. At the time of the survey the vaccination rate was furthermore still on a low level which likely influenced risk perception.

## Conclusion

Within the pandemic, the largest advantage of public transport – moving many people at once in a dense space – became its largest disadvantage and caused a great reduction in public transport usage. Even despite the changed pandemic conditions (especially in terms of scientific findings, higher vaccination rates, reopened destinations, and non-lockdown-phases), the demand for public transport has not yet recovered. The forecasts for the future of public transport also look poor: An increase in working from home is also expected beyond the duration of the pandemic. It is expected that about one-third of all appointments away from home could now be virtual. As a result of this development, it is assumed that work mobility will experience a decline of 5.5–8% in Germany (Richert et al., 2021). Only 64% of respondents said they would use public transport in the future as they did before the COVID-19 pandemic. Currie et al. (2021) predict that infection fear will remain influential in transport mode selection even after the pandemic. However, public transport is needed as a strong backbone for public services and as an important part of the transformation of the transport sector toward a sustainable mobility that is accessible and feasible for all people around the world. To achieve the climate targets and to enable mobility for all, the above-mentioned trends must be reversed as quickly as possible. Strengthening local public transport, which is indispensable for the transformation of the mobility sector, is a long way off and decrease of passengers will not reverse itself. Public transport therefore quickly needs to get used to a post-pandemic “new normal.” The study highlights that the topic of risk perception will play an important role in attracting new and former passengers within this “new normal” and reveals starting points for interventions and development toward a future pandemic-resistant public transport.

## Data Availability Statement

The raw data supporting the conclusions of this article will be made available by the authors, without undue reservation.

## Ethics Statement

Ethical review and approval was not required for the study on human participants in accordance with the local legislation and institutional requirements. Written informed consent from the participants or their legal guardian/next of kin was not required to participate in this study in accordance with the national legislation and the institutional requirements.

## Author Contributions

AH conducted the data analysis and wrote and revised the manuscript. MR conceptualized and conducted the survey and drafted the data analysis and the methods section. NS conceptualized and conducted the survey and wrote the public transport specific parts in the introduction and in the discussion. AH, MR, and NS jointly interpreted and discussed the findings. ME supported AH during the writing process by providing feedback and suggestions regarding different versions of the manuscript. CS supported AH, MR, and NS throughout the planning and conceptualization of the study and by providing feedback and suggestions regarding different versions of the manuscript. All authors contributed to the article and approved the submitted version.

## Conflict of Interest

The authors declare that the research was conducted in the absence of any commercial or financial relationships that could be construed as a potential conflict of interest.

## Publisher’s Note

All claims expressed in this article are solely those of the authors and do not necessarily represent those of their affiliated organizations, or those of the publisher, the editors and the reviewers. Any product that may be evaluated in this article, or claim that may be made by its manufacturer, is not guaranteed or endorsed by the publisher.
