# Supplementary material for: Should I Stay or Should I Go? Risk Perception and Use of Local Public Transport During the COVID-19 Pandemic
Source: Front Psychol. 2022 Jul 7;13:926539. doi: 10.3389/fpsyg.2022.926539 (PMC9306558; doi:10.3389/fpsyg.2022.926539)
Supplement: Supplementary file 1 [file Data_Sheet_1.pdf]

Supplementary Material

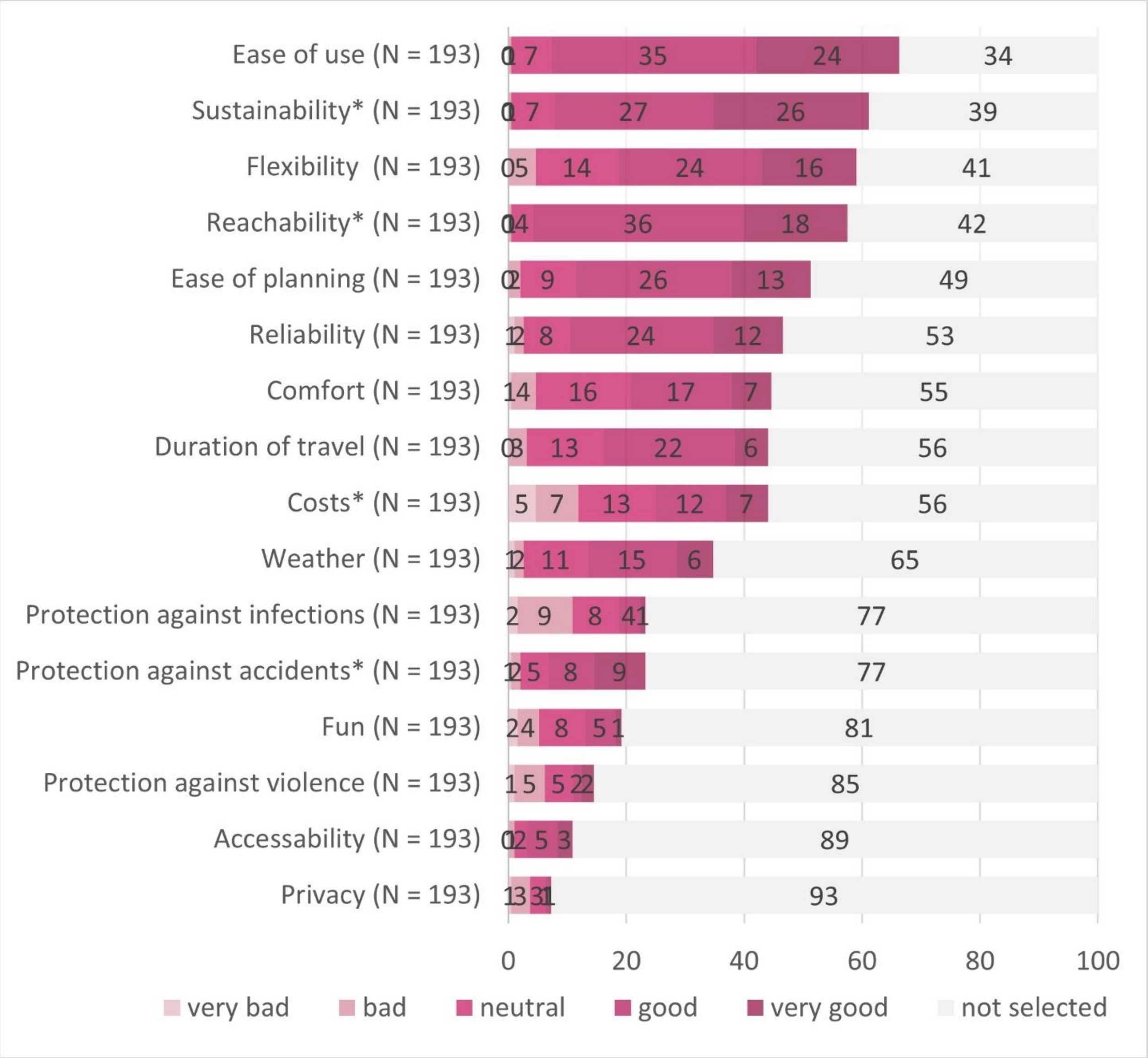

Supplementary Figure 1. *N* = 193. Loyal users' criteria of transport mode selection and their evaluation for public transport. Numbers on the bars represent percentages. * Indicates that the criterion was selected significantly more in this group often as compared to other groups with $p < .008$.

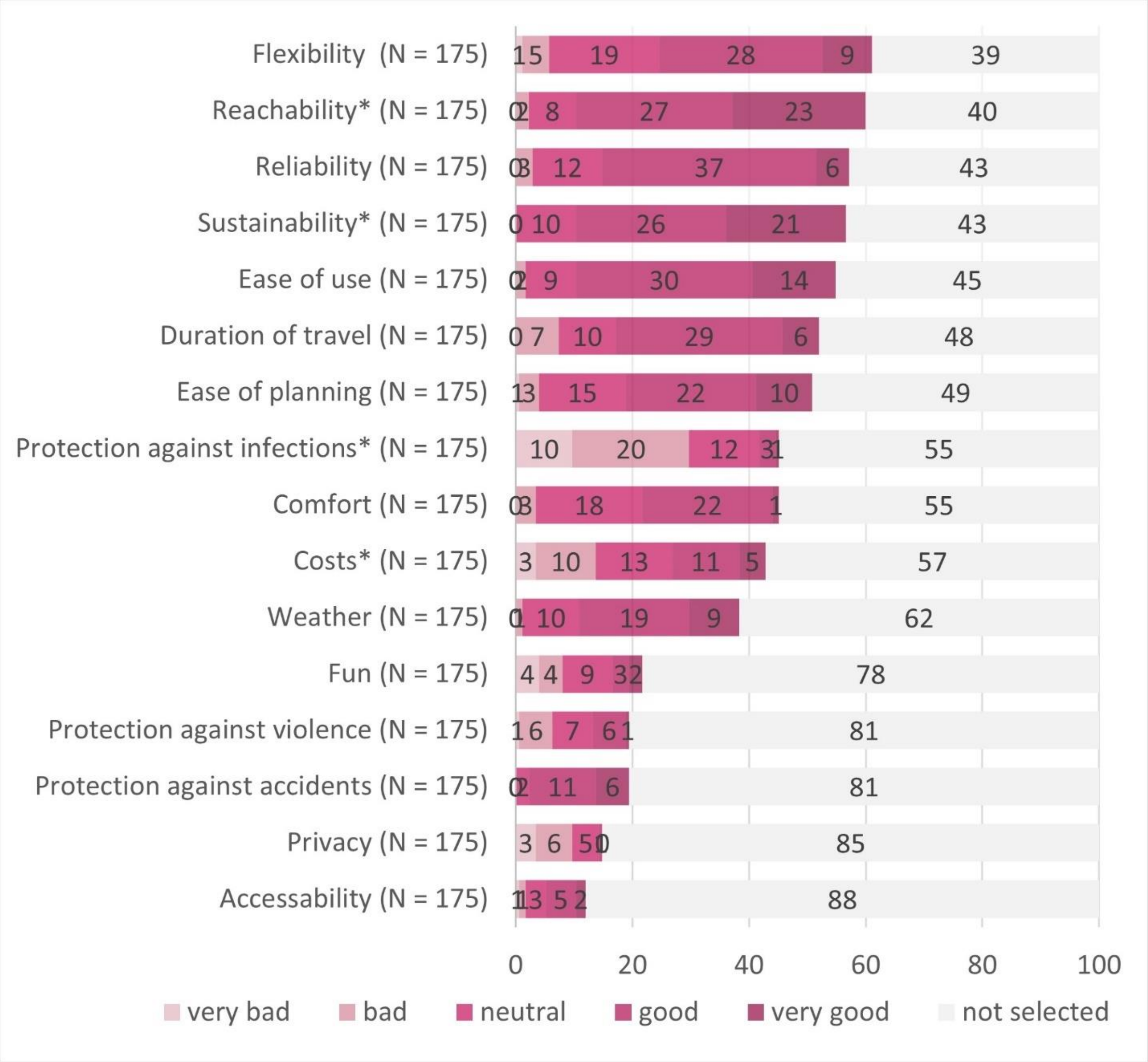

Supplementary Figure 2. *N* = 175. Reducers' criteria of transport mode selection and their evaluation for public transport. Numbers on the bars represent percentages. * Indicates that the criterion was selected significantly more often in this group as compared to other groups with $p < .008$.

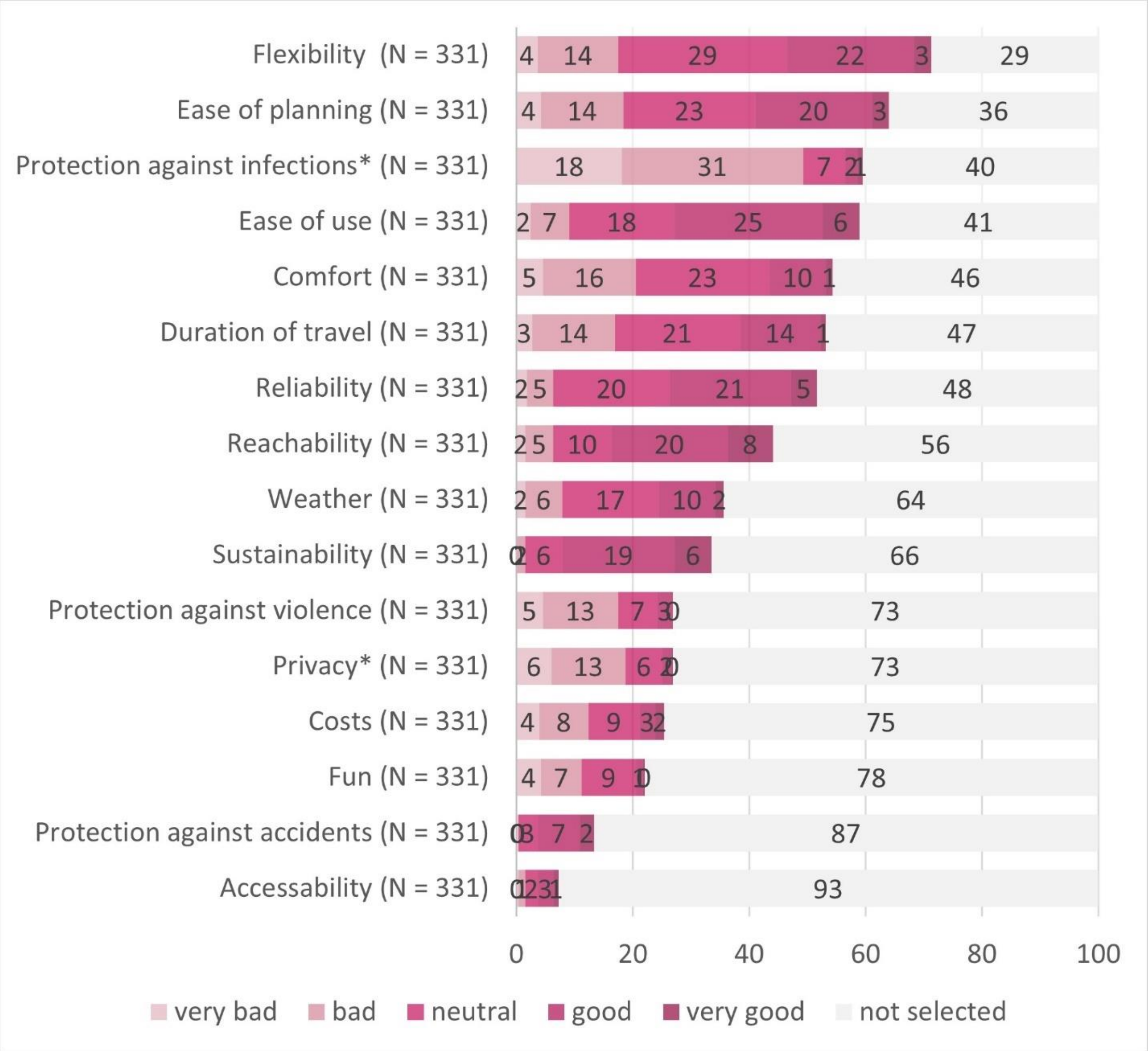

Supplementary Figure 3. *N* = 331. Pandemic dropouts' criteria of transport mode selection and their evaluation for public transport. Numbers on the bars represent percentages. * Indicates that the criterion was selected significantly more often in this group as compared to other groups with $p < .008$.

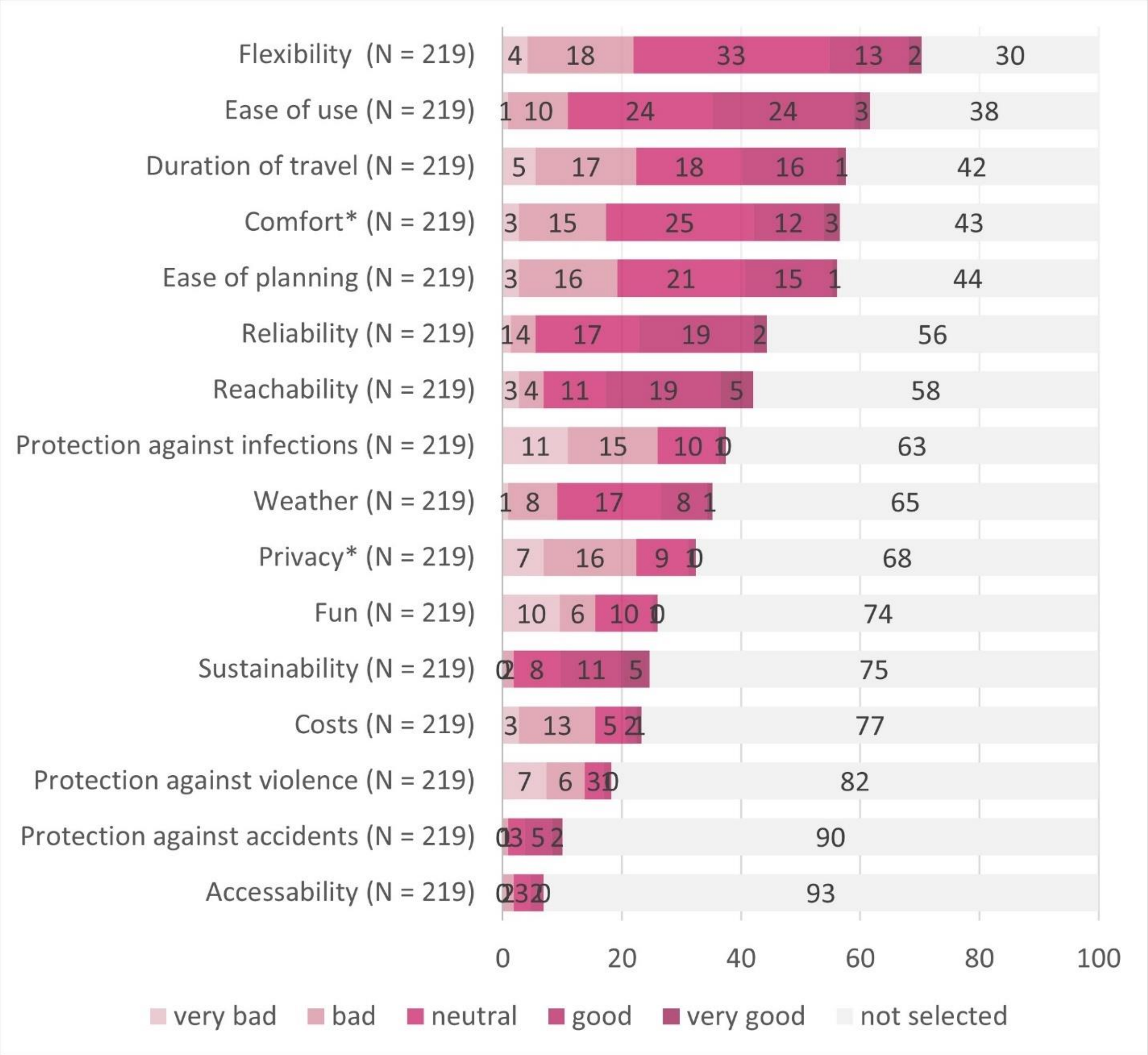

Supplementary Figure 4. *N* = 219. Non-users' criteria of transport mode selection and their evaluation for public transport in the group of non-users. Numbers on the bars represent percentages. * Indicates that the criterion was selected significantly more often in this group as compared to other groups with *p* < .008.

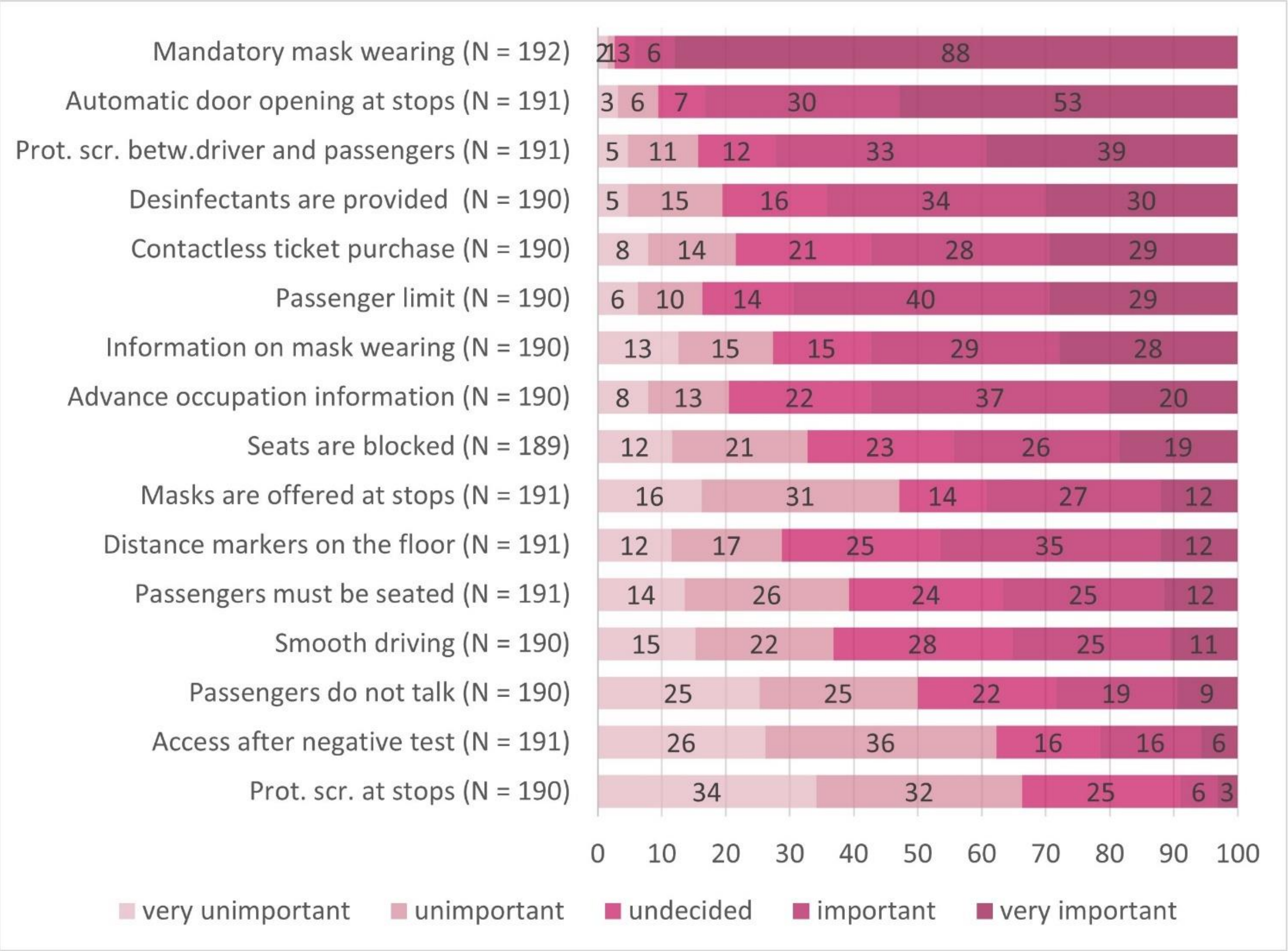

Supplementary Figure 5. *N* = 193. Loyal users' evaluation of measures against the pandemic in public transport. Prot. scr. = protective screens. Numbers on the bars represent percentages. * Indicates that the measure was evaluated significantly more important in this group as compared to other groups with *p* < .008.

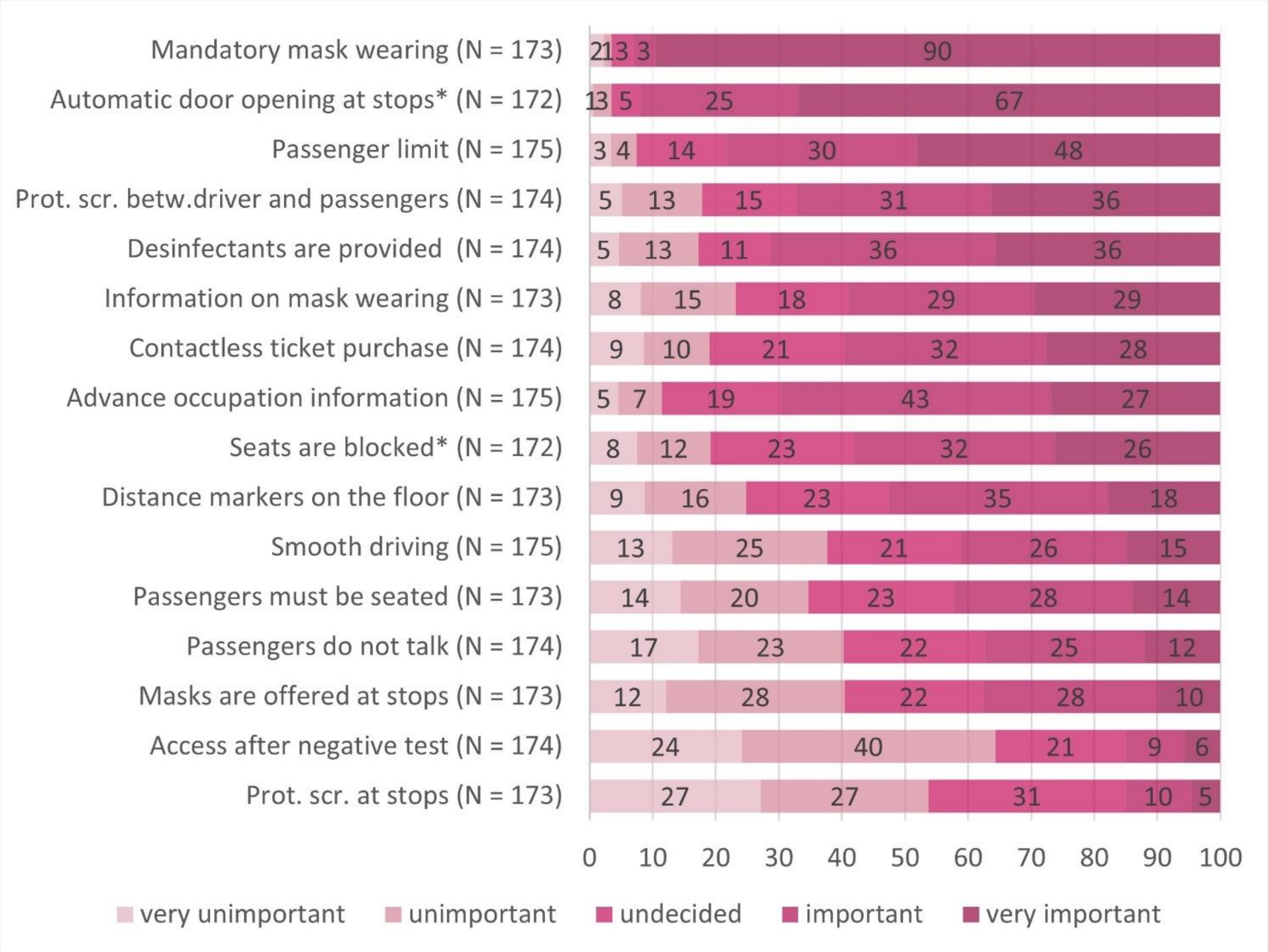

Supplementary Figure 6. *N* = 175. Reducers' evaluations of measures against the pandemic in public transport. Prot. scr. = protective screens. Numbers on the bars represent percentages. * Indicates that the measure was evaluated significantly more important in this group as compared to other groups with $p < .008$.

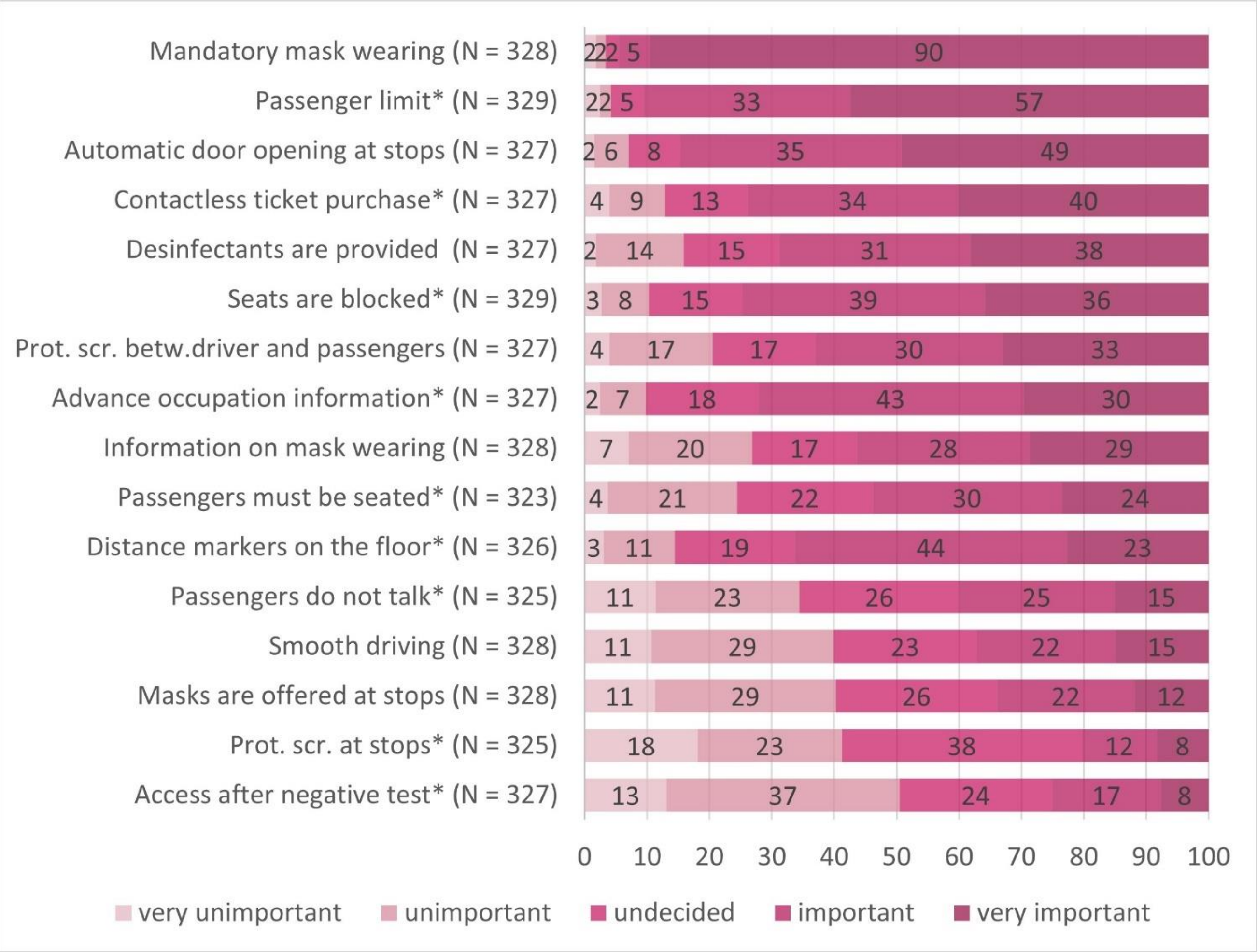

Supplementary Figure 7. *N* = 331. Pandemic dropouts' evaluation of measures against the pandemic in public transport. Prot. scr. = protective screens. Numbers on the bars represent percentages. * Indicates that the measure was evaluated significantly more important in this group as compared to other groups with $p < .008$.

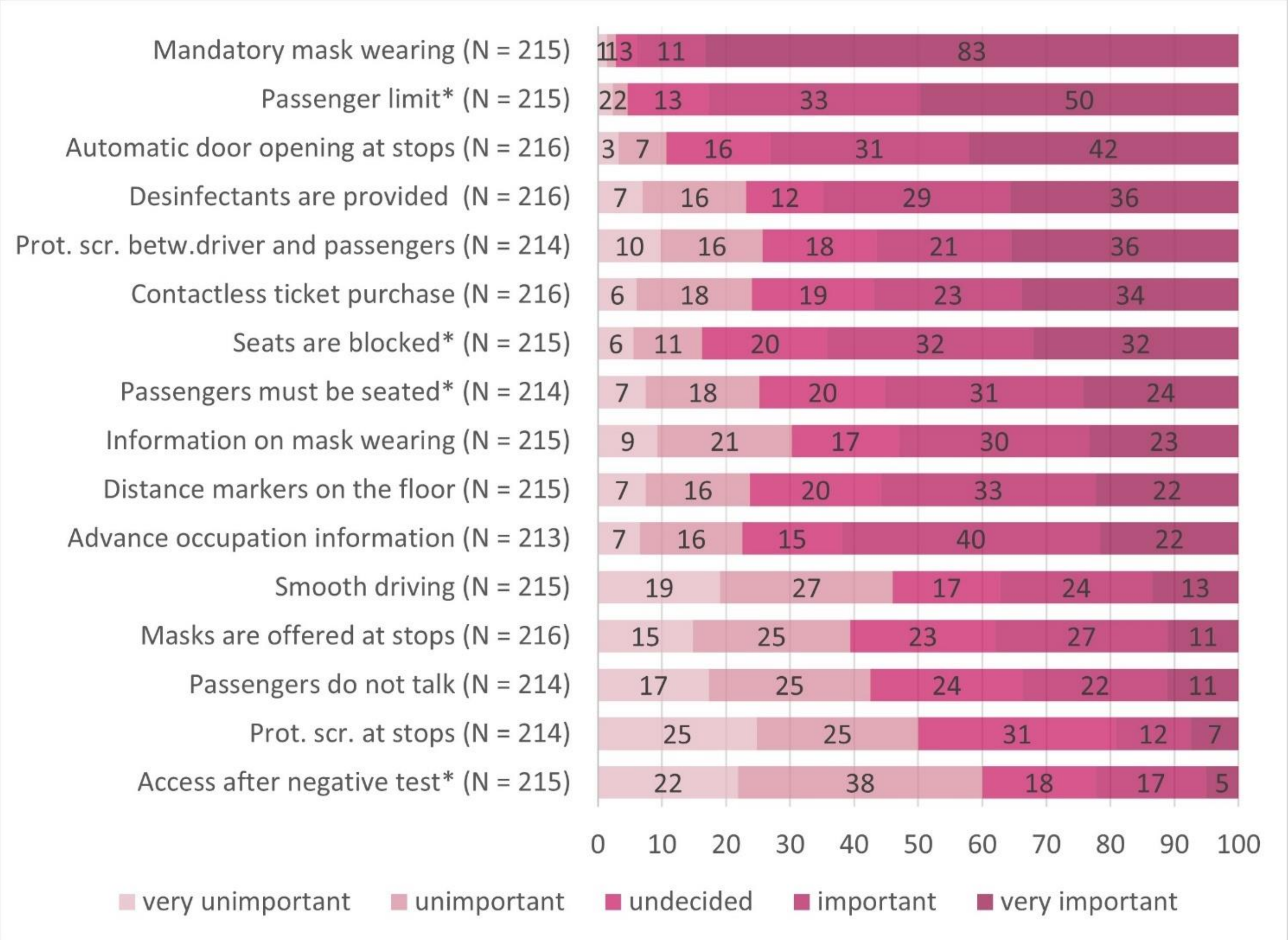

Supplementary Figure 8. *N* = 219. Non-users' evaluation of measures taken against the pandemic in public transport. Prot. scr. = protective screens. Numbers on the bars represent percentages. * Indicates that the measure was evaluated significantly more important in this group as compared to other groups with $p < .008$.
